# Empagliflozin Induced Ketosis, Upregulated IGF-1/Insulin Receptors and the Canonical Insulin Signaling Pathway in Neurons, and Decreased the Excitatory Neurotransmitter Glutamate in the Brain of Non-Diabetics

**DOI:** 10.3390/cells11213372

**Published:** 2022-10-25

**Authors:** Konstantinos I. Avgerinos, Roger J. Mullins, Michael Vreones, Maja Mustapic, Qinghua Chen, Denise Melvin, Dimitrios Kapogiannis, Josephine M. Egan

**Affiliations:** Laboratory of Clinical Investigation, Intramural Program, National Institute on Aging, NIH, Baltimore, MD 21224, USA

**Keywords:** empagliflozin, β-hydroxybutyrate, neuronal-origin extracellular vesicles, insulin signaling, magnetic resonance spectroscopy, glutamate, glutamine

## Abstract

Sodium-glucose cotransporter-2 inhibitors (SGLT2is), such as empagliflozin, lower blood glucose in type 2 diabetes mellitus and improve cardiorenal outcomes regardless of diabetes presence. Whether SGLT2is exert any effects on the brain’s metabolism has not been studied. We conducted a single-arm clinical trial to investigate the effects of once daily administration of oral empagliflozin (25 mg) for 14 days on systemic and brain metabolism in 21 non-diabetics aged 55 years old or older. Empagliflozin lowered circulating insulin and elevated β-hydroxybutyrate over 34-h periods, both following its first administration and after 14 days of daily administration, with minor alterations in glucose homeostasis. Levels of phosphorylated insulin-like growth factor-1 receptor (pIGF-1R), phosphorylated insulin receptor (pIR), phosphorylated-in-tyrosine insulin receptor substrate-1 (pY-IRS-1), and phosphorylated protein kinase B or AKT (pAKT) were increased in extracellular vesicles enriched for neuronal origin (NEVs) following the first empagliflozin administration, but not after 14 days. Our finding of IGF-1R upregulation in NEVs is promising because several post-mortem and epidemiological studies support the idea that upregulation of IGF signaling may protect against Alzheimer’s disease (AD). Moreover, our finding showing activation of insulin signaling and, in particular, the canonical pathway (pIR, pY-IRS-1, pAKT) in NEVs is important because such changes have been repeatedly associated with neuronal survival. Using brain magnetic resonance spectroscopy (MRS), we detected decreased concentrations of the excitatory neurotransmitter glutamate and its precursor glutamine after empagliflozin administration. This finding is also encouraging since glutamatergic excitotoxicity has long been implicated in AD pathology. Overall, our findings may motivate the repurposing of SGLT2is for use in AD and other, related diseases that are characterized by downregulation of IGF-1/insulin signaling in neurons and excitotoxicity.

## 1. Introduction

Sodium-glucose cotransporter-2 inhibitors (SGLT2is) were originally approved for treating type 2 diabetes mellitus (T2DM) because they lower blood glucose and HbA1c levels. These effects result from the inhibition of sodium and glucose reabsorption at the proximal tubules of the kidneys leading to their spillage in the urine [1]. The SGLT2i empagliflozin has also recently been approved for the treatment of adults suffering from heart failure with reduced ejection fraction (HFrEF). This approval came after a large multicenter trial reported a reduction in cardiovascular mortality or hospitalization due to heart failure and a reduction in kidney function decline, regardless of the presence of T2DM [2]. The mortality and cardiorenal benefits of SGLTIs may be partially explained by an induction of a state that mimics the adaptive cellular response to starvation or fasting, which involves low insulin levels, ketosis, downregulation of glycolysis, upregulation of Krebs cycle, decrease in inflammation and an increase in autophagy markers [2,3,4,5,6].

Although the fasting-like metabolic switch induced by empagliflozin could theoretically benefit any organ system, it could be particularly advantageous for the brain. Preclinical evidence shows that forms of energy restriction ameliorate common brain pathologies and are protective of cognitive function [7,8]. By potentially reducing brain exposure to chronic hyperinsulinemia that occurs even in non-diabetic states of insulin resistance [9], empagliflozin could inhibit the aggregation of pathogenic proteins such as amyloid-beta (Aβ) and Tau that underlie the pathogenesis of Alzheimer’s disease (AD) [10,11,12,13]. Additionally, inducing ketosis with empagliflozin may also have the benefit of providing the brain with ketone bodies, which are considered a more efficient energy substrate than glucose [14,15].

This study sought to investigate the metabolic changes induced in the periphery by oral empagliflozin administration to people aged 55 or older who did not have T2DM. To that end, we measured serum metabolites and plasma hormones likely to be impacted by acute and chronic empagliflozin treatment, and insulin-like growth factor-1 (IGF-1)/insulin signaling cascade mediators in plasma total extracellular vesicles (TEVs). In addition, we sought evidence that could help motivate the potential repurposing of empagliflozin for brain disorders. To that end, we investigated the brain’s metabolism by magnetic resonance spectroscopy (MRS) and interrogated the IGF-1/insulin signaling cascade in a subpopulation of plasma EVs enriched for neuronal origin (NEVs). To our knowledge, this is the first human study to explore the effects of empagliflozin on the brain’s metabolism by means of brain MRS and NEVs/TEVs isolated from plasma.

## 2. Methods

### 2.1. Trial Design and Regulatory Framework

This was a single-arm clinical trial that involved a baseline assessment (visit 1), and assessment of empagliflozin’s effects, both acutely (visit 2; first dose) and chronically (visit 3; last/14th dose). The study protocol was approved by the NIH IRB and registered in ClinicalTrials.gov (NCT03852901) before enrollment began. Subjects were recruited using multiple strategies, including, but not limited to, advertising in newspapers, flyers, the internet, NIH and NIA websites, expos and health fairs and volunteers who had expressed interest in NIA studies. All study procedures took place at the NIA Clinical Unit in Baltimore, MD, between March 2019 and November 2020. The clinical trial was conducted in compliance with the Good Clinical Practices (GCP) protocol, and all applicable regulatory requirements. Informed consent was obtained from all participants during the screening visit.

### 2.2. Participants

Included participants were non-diabetic men or women of age 55 years or older. We excluded individuals who had hypoglycemia or glucosuria in the screening labs. Individuals with significant medical conditions including class 2 obesity or more (BMI > 35 kg/m^2^), impaired kidney function (GFR < 60 mL/min/1.73 m^2^) were also excluded. For a full list of inclusion/exclusion criteria see the “study protocol” in Supplemental Othor Materials S1. We studied participants 55 years old or older since our ultimate goal is to repurpose empagliflozin (and potentially other SGLTis) as a treatment for aging-associated cognitive impairment as well as for AD and related dementias.

### 2.3. Intervention and Outcomes

Oral empagliflozin (25 mg every morning prior to breakfast) was ingested for 14 days. The primary outcome was change of serum β-hydroxybutyrate (BHB). Secondary outcomes included changes of metabolites/hormones such as plasma glucose, serum non-esterified fatty acids (NEFAs), serum acetoacetate (AcAc), plasma insulin and HOMA-IR calculated in fasting state with the formula ((insulin × glucose)/405), IGF-1, and glucagon. Additional secondary outcomes were changes of IGF-1/insulin signaling cascade molecules such as phosphorylated insulin-like growth 1 receptor (pIGF-1R), phosphorylated insulin receptor (pIR), phosphorylated-in-serine insulin receptor substrate-1 (pSer-IRS-1), phosphorylated-in-tyrosine insulin receptor substrate-1 (pY-IRS-1), (pSer-IRS-1)/(pY-IRS-1) ratio, phosphorylated extracellular signal-regulated kinase-1 (pERK-1), phosphorylated c-Jun N-terminal kinases (pJNK), phosphorylated protein kinase B (also known as AKT) (pAKT), total AKT (tAKT), and phosphorylated p38 mitogen-activated protein kinases (pp38) measured in plasma-derived total (TEVs) (to probe whole-body cell signaling) and in neuronal-origin extracellular vesicles (NEVs) (to probe neuronal-specific signaling). Other secondary outcomes included changes of brain MRS metabolites such as alanine (Ala), aspartate (Asp), creatine (Cr), phosphocreatine (PCr), gamma-aminobutyric acid (GABA), glucose (Glc), glutamine (Gln), glutamate (Glu), glycerophosphocholine (GPC), glutathione (GSH), myo-inositol (Ins), lactate (Lac), N-acetyl aspartate (NAA), N-acetylaspartylglutamate (NAAG), scyllo-inositol (Scy), taurine (Tau) phosphocholine (PCh), alanine (Ala), BHB, AcAc, and acetone (Ace). 

### 2.4. Study Procedures during Visits 

**Figure 1:**
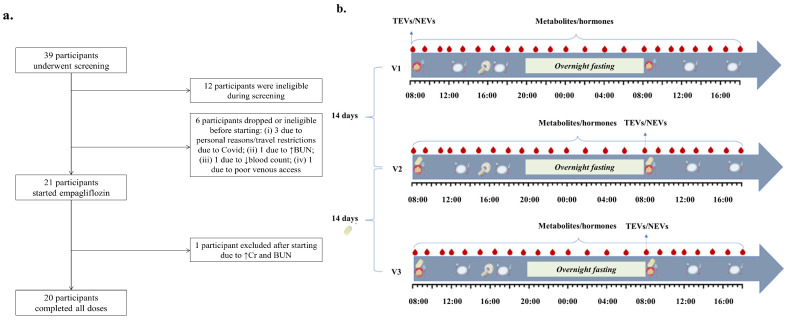
(**a**)**.** Flowchart of study population; (**b**)**.** Study procedures include: fasting blood draws for isolation of EVs (visit 1 t_o_, visit 2 t_24_ and visit 3 t_24_); empagliflozin administration from visit 2 to visit 3; breakfast, lunch and dinner; brain magnetic resonance spectroscopy (MRS); repeated blood draws for serum metabolites and plasma hormones throughout every visit [17].

Study procedures are shown in Figure 1b. Subjects were admitted for 34 h during visits 1 (baseline), 2 (first dose) and 3 (last/14th dose). Participants were admitted the evening before each visit to facilitate study procedures. After a 12-h overnight fast, participants underwent intravenous (IV) placement for a fasting blood draw (t_o_), followed by drug ingestion (visits 2 and 3) and repeated blood draws (all visits) over a 34-h window (initially every 1.5 h × 2 times, then every 1 h × 1 time, then every 1.5 h × 8 times, then every 2 h × 4 times, then every 1.5 h × 2 times, then every 1 h × 1 time, and finally every 1.5 h × 4 times). For serum collection, blood was collected into BD Vacutainer serum separation tubes (gold top, SKU: 367983). For plasma collection, blood was collected into BD Vacutainer EDTA tubes (purple top, SKU: 367856). All blood samples were immediately centrifuged at 4 °C on site and plasma and serum were stored in frozen aliquots at −80 °C in less than 1 h. Each aliquot was thawed only once. Serum metabolites and plasma hormones were measured from each blood draw (t_0_ to t_34_). TEV and NEV biomarkers were measured at fasting timepoints only (visit 1 t_o_, visit 2 t_24,_ visit 3 t_24_) to limit variation in lipoprotein concentrations that interfere with EV isolation. The standardization of the timing of blood draws, the fact that these were performed in the fasting condition, and the processing of plasma samples in < 1 h with immediate storage at −80 °C are steps that satisfy key recommendations regarding pre-analytical factors affecting EV isolation [16]. Participants had breakfast around 8.30 am (30 min after drug ingestion), lunch around 12.30 pm and dinner around 5.30 pm. MRS was performed around 4 pm on each visit. Timings of blood draws, meals and MRS were similar across all visits.

For 13 days after visit 2 (first day on medication), the participants ingested empagliflozin every morning at home about 30 min before breakfast. The participants had a continuous glucose monitoring system (FreeStyle Libre Pro 1.1.1) placed between visits to further safeguard against hypoglycemia. The end of visit 3 concluded all study procedures. 

### 2.5. Laboratory Assays

Plasma insulin was measured using an ELISA kit (Mercordia; Catalog No. 10-1113-01) [18]. Both serum BHB and AcAc were measured using colorimetric assay kits (Cayman Chemicals; Catalog No. 700190 and Sigma-Aldrich; Catalog No. MAK199, respectively), according to manufacturer’s instructions. Non-esterified fatty acids were measured by an enzymatic colorimetric method (FUJIFILM Wako Chemicals; Catalog No. 999-34691 and 995-34791) [19]. Plasma glucagon was measured using a radioimmune assay (Millipore; Catalog No. GL-32K) [18]. 

### 2.6. Isolation of Total and Neuronal Extracellular Vesicles from Blood Plasma 

An extensive description of the methodology used to isolate NEVs from plasma has been previously reported by our group [20]. Plasma aliquots (0.5 mL) were defibrinated using Thrombin (System Biosciences, Inc., Palo Alto, CA, USA). Plasma was then treated with particle precipitation solution, Exoquick (System Biosciences, Inc., Palo Alto, CA, USA). The resulting pellet containing crude EVs was resuspended in 0.7 mL of distilled water supplemented with protease and phosphatase inhibitors and was centrifuged at 400× *g* for 5 min at +4 °C to remove insoluble contaminants. After transfer to a clean tube, the supernatant was incubated with 4 µg of mouse anti-human CD171 (L1CAM; clone 5G3) biotinylated antibody (Thermo Scientific, Inc., Waltham, MA, USA) for 2 h at +4 °C, followed by incubation with 25 μL of Pierce Streptavidin Plus UltraLink Resin (Thermo Scientific, Inc., Waltham, MA, USA) for 1 h at +4 °C. After centrifugation at 800× *g* for 10 min at +4 °C, the supernatant was removed, and the pellet containing NEV-bead conjugates was further purified of soluble material by adding 500 µL of distilled water (with inhibitors) and centrifugation at 800× *g* for 10 min at +4 °C. Following removal of the supernatant, NEVs were eluted with 200 µL of 0.1 M glycine and beads were separated by centrifugation at 4500× *g* for 5 min at +4 °C. The resulting supernatant containing NEVs was transferred to a clean tube and the pH was immediately neutralized using 1 M tris-HCl. NEVs were lysed and protein was extracted using M-PER (Thermo Scientific, Inc., Waltham, MA, USA) and two freeze–thaw cycles. The NEV lysate was stored at −80 °C. A variation of the same methodology was used to capture TEVs utilizing 4 µg of a mix of antibodies against three canonical EV markers: CD9 (clone KMC8; BD Pharmingen, Inc., San Diego, CA, USA), CD63 (clone TEA3/18; Abnova, Taipei City, Taipei, Taiwan), and CD81 (clone 1.3.3.22; Ancell, Inc. Bayport, MN, USA).

To demonstrate the ultrastructural properties of NEVs, cryogenic transmission electron microscopy (Cryo-TEM) of intact NEV was performed at the Nanoscale and Microscale Research Centre of the University of Nottingham, UK. We used Holey carbon TEM grids (EM resolutions, Sheffield, UK). NEV samples were left to adsorb onto the grids (5 μL/grid) for 2 min, and then excess solution was removed using filters. The NEV samples were blotted for 1 s and were frozen in liquid ethane using a Gatan CP3 plunge freezing unit (Ametek, Leicester, UK). The frozen samples were loaded to a Tecnai G2 Spirit BioTWIN, a 20–120 kV/LaB6 Transmission Electron Microscope, with Cryo-TEM carried out with an accelerating voltage of 100 kV. We obtained the images using an inbuilt Gatan SIS Megaview IV digital camera.

Recently, we published additional evidence on the characterization of NEVs, demonstrating by WBs that L1CAM+ NEVs display the full length 220 kD characteristic band for L1CAM, which is present in brain lysate. This is unlike other sub-populations of plasma EVs that display solely the 200 kD band, a band which corresponds to soluble L1CAM [21]. Moreover, L1CAM+ NEVs contain many-fold higher L1CAM (normalized to canonical EV marker CD9) compared to other plasma EV sub-populations, suggesting the specificity of the anti-L1CAM immunoprecipitation [21]. Finally, using confocal fluorescence microscopy after double immunolabeling with L1CAM and neuronal marker VAMP2, we demonstrated the co-existence of L1CAM and VAMP2 on particles at the size range of single EVs [21]. 

### 2.7. Quantification of NEV Proteins

We quantified phosphorylated insulin signaling pathway proteins in NEV and TEV lysates using Meso Scale Discovery (MSD) electrochemiluminescence assays. pIGF-1R (Tyr1316), pIR (Tyr1150/1151), pIRS-1 (Tyr612) (Cat. # K15151C), pIRS-1 (Ser312) (Cat. # K150HLD), pJNK (Thr183/Tyr185), pp38 (Thr180/Tyr182), pERK-1/2 (Thr/Tyr: 202/204; 185/187) (Cat. # K15101D), pAkt (Ser473), and total Akt (Cat. # K15100D) were measured using the Meso QuickPlex SQ120 imager and the workbench Software 4.0 (Meso Scale Discovery, Rockville, MD, USA). Due to the absence of standards in these phospho-protein assays, we analyzed the electrochemiluminescence signal. All assays were conducted in duplicate and the mean coefficients of variation for each assay were 7.31% (IGF-1R), 7.76% (pIR), 8.79% (pTyr-IRS-1), 11.16% (pSer-IRS-1), 5.58% (pJNK), 4.69% (pp38), 12.82% (pERK1/2), 7.84% (pAKT), and 9.14% (tAKT). To assess inter-plate variability, duplicate NEV isolates from a healthy control subject were included as an internal control on each plate. The CVs for the internal control were 9.76% (IGF-1R), 5.31% (pIR), 6.86% (pTyr-IRS-1), 5.05% (pSer-IRS-1), 6.31% (pJNK), 6.37% (pp38), 3.98% (pERK1/2), 25.0% (pAKT), and 12.3% (tAKT). A correction factor was applied to the raw signal intensities of each assay to normalize inter-plate variability (internal control signal for a given plate divided by the average internal control signal of all plates). 

### 2.8. Magnetic Resonance Spectroscopy

To measure in vivo brain metabolite concentrations, single-voxel ^1^H MRS data were acquired on a Philips Achieva 3T whole-body MR scanner equipped with an 8-channel SENSE head coil. A 25 × 18 × 20 mm^3^ MRS voxel was placed within the posteromedial cortex, centered to ensure maximal coverage of bilateral precuneus, as previously undertaken [22]. Point-Resolved Spectroscopy (PRESS) with non-suppressed water reference was used to acquire metabolite concentrations, including standard water-scaled metabolites Ala, Asp, Cr, PCr, GABA, Glc, Gln, Glu, GPC, GSH, Ins, Lac, NAA, NAAG Scy, Tau, Pch, as well as the combined signals for Glu & Gln (Glx), GPC & PCh, and NAA & NAAG [23,24]. The ketone-related metabolites BHB, AcAc, and Acetone (Ace) were detected via a modified basis set provided in July of 2019 by LCModel’s developer, Dr. Stephen Provencher. This modification consisted of a supplemental scanner specific LCModel “control.txt” file that allowed us to measure BHB, AcAc, and Ace in addition to the standard set of metabolites in the basis set (Supplemental Othor Materials S2). PRESS parameters were TE = 35 msec, TR = 2000 msec, 256 averages, direct dimension bandwidth = 2 kHz, and 2048 sample points. Reliability of the measurements and fitting procedure for each metabolite was assessed using Cramer–Rao lower bounds (CRLB) [25], and line widths for water resonance were monitored for intra-subject scan reliability and were previously observed at a stable (mean ± SD) 7.3 ± 1.7 Hz.

To provide volumes of parenchymal and CSF segments within the MRS voxel, T1-weighted magnetization-prepared rapid gradient-echo (MPRAGE) images were acquired during the same session. A Turbo Field Echo acquisition sequence was used with the following parameters: TR = 6.803 ms, TE = 3.19 ms, number of excitations = 1, flip angle = 8°, acquisition matrix = 256 × 256 × 170, resolution = 1 × 1 × 1.2 mm.

### 2.9. Statistical Methods

Biofluid biomarkers were analyzed using linear mixed models with repeated measures in SPSS (Build 1.0.0.1508). For circulating metabolites/hormones assessed repeatedly over the course of 34 h during each visit, the models included participant ID as a random effect, “Visit” and “Timepoint” as repeated-measures variables, and the factors “Visit”, “Timepoint”, “Sex”, and the interaction “Visit*Sex” as terms for fixed effects. For NEV/TEV biomarkers and calculated HOMA-IR, the models included “Visit” as the repeated-measures variable. The terms for fixed effects included the factors “Visit”, “Sex”, the covariate “BMI”, and the interactions “Visit*Sex” and “Visit*BMI”. We included the interactions “Visit*Sex” and “Visit*BMI” in our models because of evidence that SGLT2 inhibitors may have different clinical effects depending on sex and BMI [26,27,28]. To correct skewness, EV biomarker measures were natural logarithm (ln)-transformed before being implemented into models.

^1^ H MRS data was processed in LCModel, an automated fitting routine that quantifies the concentration of select metabolite resonances within the MRS voxel [29]. Water-scaled metabolite concentrations (mmol/L) obtained via LCModel were then analyzed using R v.3.6.3. To minimize unreliable measurements, metabolites were censored using a strict CRLB% of <20 for the standard metabolites and a lenient exploratory < 100 for ketone metabolites (BHB, AcAc, Ace). Ala, Asp, GABA, Glc, PCh, Lac, NAAG, Scyllo, and Tau were excluded from the analysis set due to having 50% or more CRLB-censored values. Additionally, a set of weights was derived from the inverse of the CRLB for each measurement to reduce the influence of less reliable (i.e., high CRLB) measurements [30]. This ensured that more reliable (i.e., low-CRLB) measures had more impact on the statistical model than less reliable ones. We then derived the fraction of CSF (fCSF) in the MRS voxel to correct for the potential confounding effects of varying CSF volume in the precuneal voxel. To do this, we used the T_1_-weighted anatomical images (MP-RAGE) and a custom MATLAB script adapted from Partial Volume Code for Philips MRS data provided by Dr. Nia Goulden and Dr. Paul Mullins of Bangor University [31]. This script output the gray matter (GM), white matter (WM) and CSF volumes used to calculate the fractional volume of cerebrospinal fluid (fCSF = CSF_vol/_(GM_vol_ + WM_vol_ + CSF_vol_)) within the MRS voxel. Finally, we used a linear mixed effects model (nlme v.3.1) with participant ID as the random effect, MRS “Visit” (1–3) as repeated measure, each metabolite as the dependent variable, and “Visit”, “Age”, “Sex”, and “fCSF” as factors or covariates. “Age” was included in the model given strong evidence that it affects MRS metabolites [32]. This model included the aforementioned weighting term for the inverse CRLB for each metabolite, restricted maximum likelihood (REML) as the variance estimator, and first order autoregression (AR(1)) for autocorrelation.

## 3. Results

### 3.1. Participant Flow and Baseline Characteristics

Of the 39 participants that underwent screening, 18 were excluded prior to drug administration for eligibility reasons, 21 took at least one drug dose, and 20 took all drug doses completing all study visits (Figure 1a). Baseline characteristics are presented in Table 1.

### 3.2. Outcomes

In this study, we implemented repeated-measures analyses as appropriate for each outcome. Biomarkers directly assayed in plasma or serum (glucose, insulin, NEFAs, glucagon, ketone bodies) were measured multiple times over 34 h to determine their diurnal variation and relationship to meals. To demonstrate the effects of empagliflozin both acutely and after chronic administration, all outcomes were assessed at three timepoints (baseline, following the first dose, following 14 days of daily administration). We provide three different metrics (% change from baseline, F test, mean difference) for each outcome. For outcomes repeated over 34-h periods we report results for the overall pattern of repeated measurements rather than a single timepoint.

#### 3.2.1. Empagliflozin Shifted Metabolism towards a Low-Insulin, High-Glucagon State Elevating Fatty Acids, and β-hydroxybutyrate Levels

**Figure 2:**
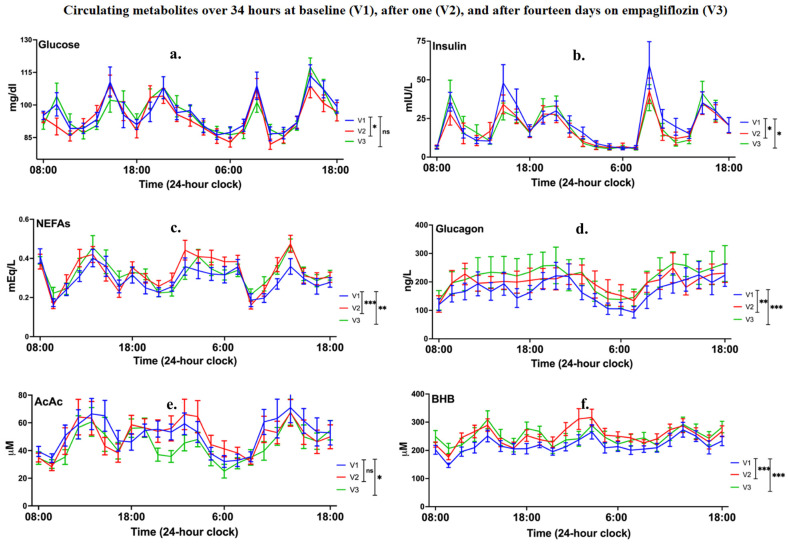
Means (SEs) of circulating metabolites and hormones ((**a**–**f**): glucose, insulin, non-esterified fatty acids (NEFAs), glucagon, acetoacetate (AcAc), β-hydroxybutyrate (BHB)) measured from blood draws over a 34-h window (22 timepoints) starting at 8 am in the morning and finishing at 6 pm the next afternoon (i.e., every 1.5 h × 2, 1 h × 1, 1.5 h × 8, 2 h × 4, 1.5 h × 2, 1 h × 1, and finally 1.5 h × 4), at baseline (visit 1 (V1)), immediately before and after the first empagliflozin dose (visit 2 (V2)), and immediately before and after the last empagliflozin dose (visit 3 (V3)). Statistical analysis involved repeated-measures linear mixed models with “Visit” as the repeated-measures variable and additional variables as factors and covariates (see Section 2.9 for details). Significance is indicated in the graphs when type III tests of fixed effects for “Visit” and pairwise comparisons between “Visits” were significant. Significance symbols ns, *, **, *** correspond to *p* > 0.05, *p* ≤ 0.05, *p* ≤ 0.01 and *p* ≤ 0.001, respectively.

Figure 2 and Table 2 show metabolites and hormone levels throughout 34-h periods for all three visits. Across all participants, BHB increased by 15% (on average over 34 h) compared with baseline after the first empagliflozin dose (F_1, 921.000_ = 54.310, *p* < 0.001; mean difference (MD) = 33.955 µM, 95% CI = 24.912, 42.997); and by 21% compared with baseline after the last dose (F_1, 902.758_ = 70.003, *p* < 0.001; MD = 40.717 µM, 95% CI = 31.166, 50.268).

The first empagliflozin dose did not change AcAc (on average over 34 h) compared with baseline (F_1, 921.000_ = 0.439, *p* = 0.508; MD = −1.281 µM; 95% CI = −5.074, 2.512), but there was an AcAc decrease by 11% compared with baseline after the last dose (F_1, 902.181_ = 9.049, *p* = 0.003; MD = −5.573 µM; 95% CI = −9.210, −1.937). Moreover, the first empagliflozin dose reduced the ratio AcAc/BHB by 16% (on average over 34 h) compared with baseline (F_1, 901.067_ = 62.430, *p* < 0.001; MD = −0.036, 95% CI = −0.045, −0.027), and the last dose reduced it by 23% compared with baseline (F_1, 878.674_ = 133.526, *p* < 0.001; MD = −0.054, 95% CI = −0.063, −0.045).

The first empagliflozin dose decreased plasma glucose by 2% (on average over 34 h) compared with baseline (F_1, 902.217_ = 3.940, *p* = 0.047; MD = −2.011 mg/dL, 95% CI = −4.000, −0.023). A significant interaction between Visit and Sex (F_1, 902.217_ = 6.704, *p* = 0.010) revealed that glucose decreased in men (F_1, 902_ = 10.461, *p* < 0.001), but remained unchanged in women (F_1, 902_ = 0.188, *p* = 0.664). Glucose values following the last drug administration were not different (on average over 34 h) compared with baseline (F_1, 884.229_ = 0.367, *p* = 0.545; MD = −0.637 mg/dL, 95% CI = −2.699, 1.426). In addition, continuous glucose monitoring confirmed that glucose levels while participants were taking empagliflozin at home were similar to baseline levels (F_1, 404_ = 0.148, *p* = 0.700). Sensitivity analysis after excluding participants with missing data yielded a similar result (F_1, 345_ = 0.485, *p* = 0.486).

Regarding insulin, the first empagliflozin dose induced a 15% decrease (on average over 34 h) compared with baseline (F_1, 902.122_ = 6.530, *p* = 0.011; MD = −3.307 mIU/L, 95% CI = −5.846, −0.767). The last dose decreased insulin by 12% (on average over 34 h) compared with baseline (F_1, 880.870_ = 3.970, *p* = 0.047; MD = −2.647 mIU/L; 95% CI = −5.255, −0.040).

The first empagliflozin dose increased glucagon levels by 12% (on average over 34 h) compared with baseline (F_1, 381_ = 7.900, *p* = 0.005; MD = 19.572 ng/L, 95% CI = 5.880, 33.263). There was an interaction between Visit and Sex (F_1, 381_ = 4.542, *p* = 0.034) showing a significant elevation in men (F_1, 381_ = 27.476, *p* < 0.001), but not women (F_1, 381_ = 0.148, *p* = 0.700). Similarly, the last dose increased glucagon by 16% (on average over 34 h) compared with baseline (F_1, 374.045_ = 10.636, *p* = 0.001; MD = 25.416 ng/L; 95% CI = 10.092, 40.741). An interaction between Visit and Sex was also found after the last dose (F_1, 374.045_ = 14.925, *p* < 0.001), showing that glucagon increased in men (F_1, 374_ = 61.711, *p* < 0.001), but did not change in women (F_1, 374_ = 0.114, *p* = 0.736).

The first empagliflozin dose resulted in increased NEFAs by 12% (on average over 34 h) compared with baseline (F_1, 902.094_ = 15.986, *p* < 0.001; MD = 0.035 mEq/L, 95% CI = 0.018, 0.053). Similarly, the last dose increased NEFAs by 9% (on average over 34 h) compared with baseline (F_1, 880.629_ = 8.782, *p* = 0.003; MD = 0.027 mEq/L; 95% CI = 0.009, 0.044).

#### 3.2.2. Empagliflozin Acutely Upregulated IGF-1/Insulin Cascade-Associated Proteins in Plasma-Derived NEVs, but Not in TEVs

Cryo-TEM pictures of NEV preparations demonstrate multiple examples of small and medium size EVs with typical morphology (single membranous nanoparticles) and size distribution. These pictures are highly consistent with the isolation of a mixed population of exosomes and microvesicles (Supplemental Figure S1; scale bars included for reference).

**Figure 3:**
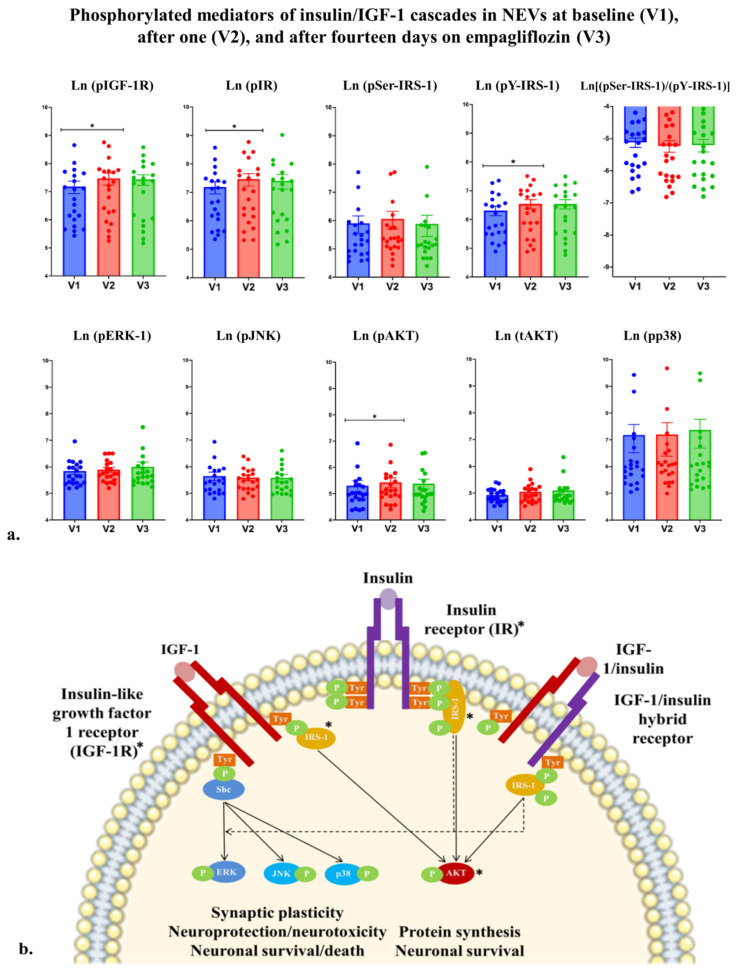
(**a**). means (SE) of phosphorylated (*p*) and total (t) levels of IGF-1/insulin cascade-associated proteins measured in extracellular vesicles enriched for neuronal origin (NEVs) at baseline (visit 1 t_0_ (V1)), 24 h after the first dose (visit 2 t_24_ (V2)), and 24 h after the last dose (visit 3 t_24_ (V3)). Statistical analysis involved repeated-measures linear mixed models with “Visit” as the repeated-measures variable and additional variables as factors and covariates (see Section 2.9 for details). Significance is indicated as *, corresponding to *p* ≤ 0.05. (**b**). IGF-1/insulin signaling in neurons reflecting acute biomarker changes detected in NEVs. Significant increases are marked with * [17].

Figure 3 and Table 3 show phosphorylated levels of IGF-1/insulin cascade mediators measured in TEVs and NEVs at visit 1 (t_o_), visit 2 (t_24_), and visit 3 (t_24_). No changes of pIGF-1R levels were identified in TEVs after the first or last empagliflozin doses compared with baseline. However, levels of pIGF-1R in NEVs significantly increased after the first empagliflozin dose compared with baseline (F _1, 18_ = 7.706, *p* = 0.012; MD = 0.247; 95% CI = 0.075, 0.419). In addition, there was a significant interaction between Visit and BMI (F_1, 18_ = 9.482, *p* = 0.06), with lower-than-mean BMI being associated with no change in NEV pIGF-1R levels (F_1, 18_ = 0.013, *p* = 0.911), mean BMI being associated with NEVs pIGF-1R increase (F_1, 18_ = 9.076, *p* = 0.007), and higher-than-mean BMI being associated with a sharper NEV pIGF-1R increase (F_1, 18_ = 18.381, *p* < 0.001). After the last dose, NEV pIGF-1R levels were similar to baseline (F_1, 17.267_ = 3.342, *p* = 0.085; MD = 0.229; 95% CI = 0.023, 0.434).

No change in TEV pIR was found after the first or last empagliflozin dose compared with baseline. However, levels of NEV pIR significantly increased after the first empagliflozin dose compared with baseline (F _1, 18_ = 7.905, *p* = 0.012; MD = 0.196; 95% CI = 0.036, 0.357). In addition, there was a significant interaction between Visit and BMI (F_1, 18_ = 9.435, *p* = 0.007) with lower-than-mean BMI being associated with no NEV pIR change (F_1, 18_ = 0.177, *p* = 0.679), mean BMI being associated with an NEV pIR increase (F_1, 18_ = 6.582, *p* = 0.019), and higher-than-mean BMI being associated with a sharper NEV pIR increase (F_1, 18_ = 15.778, *p* < 0.001). After the last dose, NEV pIR levels were similar to baseline (F_1, 17.465_ = 1.151, *p* = 0.298; MD = 1.20; 95% CI = −0.167, 0.406).

No change was found for TEV pY-IRS-1 after the first or last empagliflozin dose. However, the first empagliflozin dose increased NEV pY-IRS-1 (F_1, 18_ = 9.934, *p* = 0.006; MD = 0.174; 95% CI = 0.065, 0.284). In addition, there was a significant interaction between Visit and BMI (F_1, 18_ = 12.168, *p* = 0.003) with lower-than-mean BMI being associated with no NEV pY-IRS-1 change (F_1, 18_ = 0.003, *p* = 0.859), mean BMI being associated with an NEV pY-IRS-1 increase (F_1, 18_ = 11.149, *p* = 0.004), and higher-than-mean BMI being associated with a sharp NEV pY-IRS-1 increase (F_1, 18_ = 23.096, *p* < 0.001). After the last dose, NEV pY-IRS-1 levels were not different compared with baseline (F_1, 17.235_ = 0.832, *p* = 0.374; MD = 0.140; 95% CI = −0.043, 0.323).

No changes were found for pAKT measured in TEVs after the first or last dose, compared with baseline. Levels of pAKT in NEVs significantly increased after the first empagliflozin dose compared with baseline (F _1, 18_ = 5.650, *p* = 0.029; MD = 0.154; 95% CI = 0.039, 0.269). In addition, there was a significant interaction between Visit and BMI (F_1, 18_ = 7.075, *p* = 0.016) with lower-than-mean BMI being associated with no NEV pAKT change (F_1, 18_ = 0.003, *p* = 0.958), mean BMI being associated with an NEV pAKT increase (F_1, 18_ = 7.950, *p* = 0.011), and higher-than-mean BMI being associated with a sharper NEV pAKT increase (F_1, 18_ = 14.854, *p* = 0.001). After the last dose, NEV pAKT levels were not different compared with baseline (F_1, 17.403_ = 0.105, *p* = 0.749; MD = 0.088; 95% CI = −0.070, 0.247). However, results regarding pAKT should be interpreted with caution as its CV for internal control exceeded 25%.

No changes were identified for pSer-IRS-1, tAKT, pJNK, pp38, pERK1/2 in TEVs or NEVs after the first or last empagliflozin dose, compared with baseline.

#### 3.2.3. Chronic Empagliflozin Administration Decreased Brain Glutamate and Glutamine Concentrations

**Figure 4:**
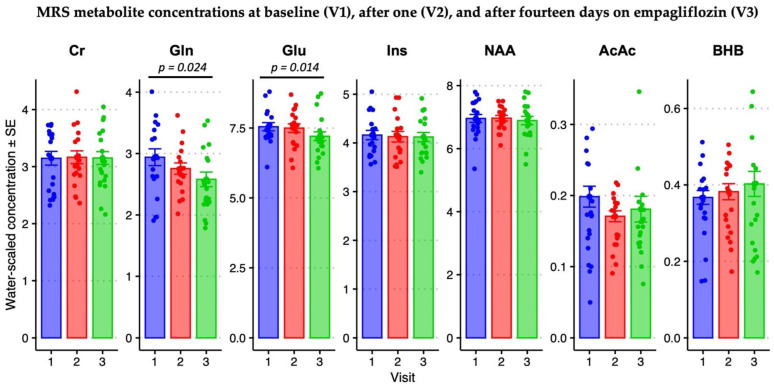
Select precuneal water-scaled MRS metabolite concentrations (mmol/L) over the three study visits. Standard error bars indicate the water-scaled concentration of Creatine (Cr), Glutamine (Gln), myo-Inositol (Ins), N-acetyl aspartate (NAA), Acetoacetate (AcAc) and beta-hydroxybutyrate (BHB). Statistical analysis involved repeated-measures linear mixed models with “Visit” as the repeated-measures variable and concentration as the dependent variable. Additional factors and covariates are detailed in the Statistical Methods Section 2.9. Bars and error bars are shown for baseline (visit 1 t_0_ (1)), 24 h after the first dose (visit 2 t_24_ (2)), and 24 h after the last dose (visit 3 t_24_ (3)). Significant results for the omnibus test across all three visits are indicated by a black horizontal bar above the individual plot, with the *p*-value above.

Figure 4 and Table 4 show details on water-scaled ^1^H MRS metabolite concentrations (mmol/L) over the course of the study. The concentrations of Gln (F_2,34_ = 4.15, *p* = 0.024) and Glu (F_2,35_ = 4.82, *p* = 0.014) decreased over the three visits, as did the combined Glu & Gln (or “Glx”) measure (F_2,35_ = 7.33, *p* = 0.002). No other metabolites changed significantly over the course of the study. Post-hoc pairwise comparisons via the least significant difference method (LSD) show a 9% Gln decrease after the last empagliflozin dose compared with baseline (*p* = 0.036, MD = −0.270 mmol/L, 95% CI = −0.522, −0.0188). A 5% decrease by the last dose was also evident for the combination of Glu and Gln (*p* = 0.010, MD = −0.545 mmol/L, 95% CI = −0.951, −0.138) but fell short of significance for Glu itself (*p* = 0.051, MD = −0.231 mmol/L, 95% CI = −0.462, 0.001). There were no significant pairwise differences between the first dose and baseline.

### 3.3. Adverse Events

Except for one participant (1/21) who was stopped from the study due to mild Cr elevation, no other adverse event was observed.

## 4. Discussion

In this clinical study, we found that empagliflozin decreased insulin, increased glucagon, and increased NEFAs and BHB blood levels in individuals without diabetes [33]. While the first dose of empagliflozin lowered circulating glucose levels, after 14 days of daily empagliflozin (visit 3), glucose levels were similar to baseline (visit 1). These findings suggest a rapid diminution of the glucose-lowering effect of empagliflozin in individuals without diabetes and a restoration of glucose homeostasis, which facilitates any potential repurposing of the drug for medical indications besides diabetes. Interestingly, we found that empagliflozin acutely elevated pIGF-1R, pIR and common downstream mediators of IGF and insulin signaling such as pY-IRS-1 and pAKT in NEVs but not in TEVs, indicating a neuronal-specific upregulation of these pathways. Although no elevation of ketone bodies was directly observed in the brain using MRS, empagliflozin reduced the excitatory neurotransmitter glutamate and its precursor glutamine, metabolites that have been shown to respond to ketogenic interventions.

Overall, empagliflozin induced metabolic changes that overlap with those of fasting and calorie restriction, such as a metabolic shift towards lipolysis and ketogenesis [3,33,34]. Also analogous to calorie restriction, chronic empagliflozin administration decreased circulating insulin levels without significantly altering glucose homeostasis [34,35]. Empagliflozin’s insulin-reducing capability is of great importance for neurodegenerative diseases, as chronically elevated insulin levels downregulate autophagy and inhibit clearance of abnormal proteins [4,36,37]. Evidence from other clinical studies suggests that empagliflozin induces beneficial cellular responses regardless of presence or absence of hyperglycemia [2,4]. Such benefits may be due to empagliflozin-induced activation of *SIRT1* and *AMPK*, potentially as a response to glucose loss in the urine, which constitutes a form of nutrient deprivation. Activation of *SIRT1* and *AMPK* has been associated with increased autophagy and reduced inflammation and oxidative stress. These downstream effects may promote cellular resilience in the brain and other organs [4,38,39].

We showed that empagliflozin elevated circulating BHB levels after acute and chronic dosing, in agreement with a previous study of empagliflozin that showed a trend towards BHB elevation in a non-diabetic population [33]. Accumulating evidence suggests that ketones are an efficient alternative to glucose as an energy fuel, and so may be potentially useful as an intervention in chronic neurodegenerative disorders such as AD, in which the brain has a decreased ability to efficiently utilize glucose [14,15,40]. Therefore, BHB elevation by empagliflozin may be beneficial for such disorders. Interestingly, despite the observed BHB increase, AcAc was decreased and the ratio AcAc/BHB was also decreased. The decreased AcAc/BHB ratio may reflect increased conversion of AcAc to BHB, typically associated with an elevated NAD^+^/NADH ratio. Evidence from animal models indicates that NAD^+^ induces enzymes involved in promoting neuronal health, such as sirtuins, and therefore a potential NAD^+^ increase by empagliflozin would be beneficial in AD and other neurodegenerative disorders [41].

Following the first dose, empagliflozin induced significant elevations of pIGF-1R, pIR, and common downstream mediators of the IGF and insulin signaling pathways such as pY-IRS-1 and pAKT (with the caveat of high CV for the internal control) in NEVs. These changes indicate an activation of the IGF-1 and insulin signaling pathways in neurons. No NEV biomarker differences were observed after the last empagliflozin dose compared with baseline, perhaps due to a potential adaptation to repeated dosing. Our findings on the acute elevation of proteins of the IGF-1 and insulin signaling pathways detected in NEVs are important because these NEV biomarkers have been linked to AD diagnosis [42], age-related cognitive decline [43], grey matter volume and volume of white matter hyperintensities [44]. Post-mortem studies, have shown that AD brains are characterized by decreased IGF-1R and IGF-1 mRNA levels (that worsen with increasing Braak stage) [45], as well as decreased IRS-1 levels [46]. In the Framingham study, decreased serum IGF-1 levels predicted the development of future AD, providing additional evidence for the involvement of IGF-1/IGF-1R signaling disturbances in AD [47]. In contrast, evidence mainly from animal models indicates that inhibition of IGF-1R is associated with longevity and neuronal resilience to AD pathologies [48,49,50]. Therefore, the effects of empagliflozin on the IGF-1 signaling cascade and its interplay with AD pathologies requires further investigation in vitro and in vivo. Of note, empagliflozin induced elevations of proteins of the canonical pathway of insulin signaling (pIR, pY-IRS-1, pAKT). Interestingly, activation of the canonical pathway is known to promote neuronal survival [51,52]. Empagliflozin did not alter proteins of the alternative pathway (i.e., (i) ERK which has been associated with both neuronal survival (acute activation) and death (chronic activation), and is additionally involved in synaptic plasticity [53,54,55,56]; (ii) JNK which has been associated with neuronal death and synaptic plasticity [57]; and (iii) p38 which has been associated with neurotoxicity or neuroprotection, depending on the p38 subtype activated or neuron type involved [58]). Prior NEV studies have shown that drugs with different mechanisms of action can affect the cascade downstream of IRS-1 differently; for example, exenatide acts on AKT [59], but infliximab acts on JNK, p38, ERK1/2 [60].

The primary MRS finding was a significant decrease in brain glutamate and glutamine after empagliflozin administration, which is consistent with earlier findings in ketogenic intervention studies. BHB has been shown to decrease glutamate availability in vitro, ostensibly due to reduced malate-aspartate shuttle activity in neurons reliant on BHB [61]. This finding is also consistent with the effect of ketogenic diets on glutamine levels as a treatment for glioblastoma, in which ketogenic diets inhibit both glycolysis and glutaminolysis [62]. This decrease in glutamate is also interesting in the context of both epilepsy [63] and AD [64,65], in which glutamatergic excitotoxicity has long been implicated in their respective pathological cascades. In AD, the presence of Tau tangles and Aβ plaques appears to exacerbate the effects of glutamatergic excitotoxicity [66,67]. Unfortunately, the ketone metabolites (BHB, AcAc, Ace) were difficult to detect reliably in this study due to their low concentration.

The main limitation of this study was its small sample size. Nevertheless, the study had many strengths. First, it included repeated assessments of metabolites and hormones over 34 h to provide detailed time courses of their change. Second, we gained insight into how drug effects may change with meals, the sleep–wake cycle, and duration of treatment. Third, our study included equal numbers of men and women, and therefore our findings have added value, demonstrating that both sexes have increased circulating BHB, upregulated IGF-1/insulin receptors and proteins of the canonical pathway of insulin signaling in neurons, and decreased brain Gln and Glu. Fourth, our study population reflects the age at which this class of drugs may be studied as potential therapies for cognitive impairment. Additional strengths were the integration of multiple complementary methodologies to probe brain effects, particularly NEV isolation, and brain MRS. This enabled us to derive a plethora of biologically relevant outcomes, including circulating metabolites and hormones, brain metabolites, and EV biomarkers. This study also distinguished between neuronal-specific and systemic effects on biomarkers of the IGF-1/insulin cascade by isolating different EV populations.

Overall, our findings confirm the known ketogenic effect of empagliflozin and additionally provide novel evidence that empagliflozin potentially promotes neuronal survival through activation of the canonical pathway of insulin signaling and may act as a neuroprotectant through upregulation of IGF-1R (based on studying NEVs). We also showed that empagliflozin decreases potentially harmful excitatory neurotransmission in the brain by decreasing glutamate and glutamine (based on brain MRS). These neuronal and brain effects may be beneficial in relevance to AD and related dementias and motivate the repurposing of empagliflozin for these disorders. Future double-blind placebo-controlled randomized clinical trials that may include these and additional biomarkers, as well as cognitive and functional brain outcomes, are needed to test this hypothesis.

## Figures and Tables

**Table 1:** Baseline demographic and laboratory characteristics.

Sample Size (n)	21
Sex (F/M)	11/10
Age (years)	62.14 (6.91)
BMI (kg/m^2^)	27.15 (2.97)
Fasting glucose (mg/dL)	95.19 (8.79)
Fasting Insulin (mIU/L)	6.32 (4.76)
HOMA-IR	1.54 (1.30)
Glucagon (ng/L)	120.04 (63.03)
NEFAs (mEq/L)	0.41 (0.17)
BHB (µM)	202.38 (72.86)
AcAc (µM)	39.40 (16.65)
Continuous variables are reported as means (SDs)	

**Table 2:** Serum metabolites and plasma hormones measured in 34-h periods at baseline (V1), after one dose (V2), and after multiple (V3) empagliflozin doses.

Circulating Metabolite/ Hormone/ Other	Visits Compared	Type III Tests of Fixed Effects				Pairwise Comparisons (Least Significant Difference)					
		Num df	Den df	F	Sig.	Mean Difference	Std. Error	df	Sig.	95% Confidence Interval	
										Lower Bound	Upper Bound
Glucose	V2 vs. V1	1	902.217	3.940	**0.047**	−2.011	1.013	902.217	**0.047**	−4.000	−0.023
	V3 vs. V1	1	884.229	0.367	0.545	−0.637	1.051	884.229	0.545	−2.699	1.426
Insulin	V2 vs. V1	1	902.122	6.530	**0.011**	−3.307	1.294	902.122	**0.011**	−5.846	−0.767
	V3 vs. V1	1	880.870	3.970	**0.047**	−2.647	1.329	880.870	**0.047**	−5.255	−0.040
IGF-1	V2 vs. V1	1	18.000	0.000	0.993	−0.001	0.059	18.000	0.993	−0.124	0.123
	V3 vs. V1	1	18.000	0.212	0.651	−0.023	0.051	18.000	0.651	−0.131	0.084
HOMA-IR	V2 vs. V1	1	19.000	0.520	0.480	0.077	0.107	19.000	0.480	−0.147	0.302
	V3 vs. V1	1	18.224	2.588	0.125	−0.242	0.151	18.224	0.125	−0.559	0.074
Glucagon	V2 vs. V1	1	381.000	7.900	**0.005**	19.572	6.963	381.000	**0.005**	5.880	33.263
	V3 vs. V1	1	374.045	10.636	**0.001**	25.416	7.793	0.001	**0.001**	10.092	40.741
NEFAs	V2 vs. V1	1	902.094	15.986	**<0.001**	0.035	0.009	902.094	**<0.001**	0.018	0.053
	V3 vs. V1	1	880.629	8.782	**0.003**	0.027	0.009	880.629	**0.003**	0.009	0.044
AcAc	V2 vs. V1	1	921.000	0.439	0.508	−1.281	1.933	921.000	0.508	−5.074	2.512
	V3 vs. V1	1	902.181	9.049	**0.003**	−5.573	1.853	902.181	**0.003**	−9.210	−1.937
BHB	V2 vs. V1	1	921.000	54.310	**<0.001**	33.955	4.607	921.000	**<0.001**	24.912	42.997
	V3 vs. V1	1	902.758	70.003	**<0.001**	40.717	4.866	902.758	**<0.001**	31.166	50.268
AcAc/BHB	V2 vs. V1	1	901.067	62.430	**<0.001**	−0.036	0.005	901.067	**<0.001**	−0.045	−0.027
	V3 vs. V1	1	878.674	133.526	**<0.001**	−0.054	0.005	878.674	**<0.001**	−0.063	−0.045

**Table 3:** IGF-1/insulin cascade-associated proteins measured in total EVs (TEVs) and EVs enriched for neuronal origin (NEVs) at baseline (V1), after one dose (V2), and after multiple (V3) empagliflozin doses.

			Type III Tests of Fixed Effects				Pairwise Comparisons (Least Significant Difference)					
Biomarker	Type of Exosomes	Dosing	Num df	Den df	F	Sig.	Mean Difference	Std. Error	df	Sig.	95% Confidence Interval	
											Lower Bound	Upper Bound
pIGF-1R	TEVs	V2 vs. V1	1	18.000	0.410	0.530	0.429	0.164	18.000	**0.018**	0.084	0.773
		V3 vs. V1	1	18.711	0.583	0.455	0.263	0.236	17.820	0.278	−0.232	0.759
	NEVs	V2 vs. V1	1	18.000	7.706	**0.012**	0.247	0.082	18.000	**0.007**	0.075	0.419
		V3 vs. V1	1	17.267	3.342	0.085	0.229	0.098	18.087	**0.031**	0.023	0.434
pIR	TEVs	V2 vs. V1	1	18.000	0.469	0.502	0.088	0.163	18.000	0.595	−0.254	0.430
		V3 vs. V1	1	17.896	0.094	0.763	0.016	0.149	17.772	0.913	−0.296	0.329
	NEVs	V2 vs. V1	1	18.000	7.905	**0.012**	0.196	0.076	18.000	**0.019**	0.036	0.357
		V3 vs. V1	1	17.465	1.151	0.298	0.120	0.136	17.820	0.391	−0.167	0.406
pSer- IRS-1	TEVs	V2 vs. V1	1	18.000	0.515	0.482	0.066	0.107	18.000	0.545	−0.159	0.290
		V3 vs. V1	1	17.302	0.378	0.547	−0.059	0.086	17.942	0.498	−0.240	0.121
	NEVs	V2 vs. V1	1	18.000	0.004	0.948	0.117	0.098	18.000	0.245	−0.088	0.322
		V3 vs. V1	1	17.500	3.100	0.096	−0.081	0.116	17.704	0.493	−0.325	0.163
pY-IRS-1	TEVs	V2 vs. V1	1	18.000	0.175	0.681	0.025	0.130	18.000	0.848	−0.249	0.299
		V3 vs. V1	1	17.832	0.199	0.661	−0.071	0.126	17.833	0.578	−0.335	0.193
	NEVs	V2 vs. V1	1	18.000	9.934	**0.006**	0.174	0.052	18.000	**0.004**	0.065	0.284
		V3 vs. V1	1	17.235	0.832	0.374	0.140	0.087	17.916	0.125	−0.043	0.323
(pSer-IRS-1)/ (pY-IRS-1)	TEVs	V2 vs. V1	1	18.000	0.060	0.809	0.040	0.090	18.000	0.659	−0.149	0.230
		V3 vs. V1	1	17.737	0.000	0.997	0.010	0.109	17.671	0.930	−0.219	0.238
	NEVs	V2 vs. V1	1	18.000	2.200	0.155	−0.057	0.115	18.000	0.627	−0.299	0.185
		V3 vs. V1	1	17.375	6.732	**0.019**	−0.222	0.111	17.316	0.062	−0.456	0.013
pERK-1/2	TEVs	V2 vs. V1	1	18.000	0.591	0.452	0.294	0.117	18.000	**0.022**	0.048	0.540
		V3 vs. V1	1	18.147	1.184	0.291	0.090	0.124	17.552	0.477	−0.171	0.352
	NEVs	V2 vs. V1	1	18.000	0.481	0.497	0.082	0.085	18.000	0.351	−0.098	0.261
		V3 vs. V1	1	17.039	4.284	0.054	0.062	0.072	17.098	0.403	−0.090	0.213
pJNK	TEVs	V2 vs. V1	1	18.000	0.777	0.390	0.228	0.119	18.000	0.071	−0.022	0.477
		V3 vs. V1	1	18.956	0.579	0.456	−0.012	0.120	18.009	0.922	−0.265	0.241
	NEVs	V2 vs. V1	1	18.000	0.147	0.706	0.015	0.128	18.000	0.908	−0.254	0.284
		V3 vs. V1	1	17.554	6.694	**0.019**	−0.023	0.111	16.921	0.837	−0.257	0.211
pAKT	TEVs	V2 vs. V1	1	18.000	0.317	0.580	0.061	0.088	18.000	0.496	−0.123	0.245
		V3 vs. V1	1	17.816	0.892	0.357	−0.029	0.098	17.675	0.772	−0.235	0.177
	NEVs	V2 vs. V1	1	18.000	5.650	**0.029**	0.154	0.055	18.000	**0.011**	0.039	0.269
		V3 vs. V1	1	17.403	0.105	0.749	0.088	0.075	17.801	0.256	−0.070	0.247
tAKT	TEVs	V2 vs. V1	1	18.000	0.309	0.585	0.106	0.096	18.000	0.281	−0.095	0.307
		V3 vs. V1	1	18.670	1.126	0.302	−0.021	0.117	18.023	0.857	−0.267	0.225
	NEVs	V2 vs. V1	1	18.000	1.385	0.255	0.089	0.064	18.000	0.177	−0.044	0.223
		V3 vs. V1	1	18.197	2.108	0.164	0.075	0.072	17.707	0.310	−0.076	0.226
pp38	TEVs	V2 vs. V1	1	18.000	0.695	0.416	0.200	0.106	18.000	0.076	−0.023	0.423
		V3 vs. V1	1	17.071	0.383	0.544	0.067	0.140	17.844	0.639	−0.228	0.361
	NEVs	V2 vs. V1	1	18.000	0.558	0.465	0.037	0.086	18.000	0.674	−0.144	0.218
		V3 vs. V1	1	15.292	6.202	**0.025**	0.054	0.064	16.699	0.411	−0.081	0.188

**Table 4:** MRS metabolite reliability measures, descriptive statistics, and omnibus statistical results over the baseline (V1), after one dose (V2), and after multiple (V3) empagliflozin doses.

Metabolite	CRLB%	Visit 1	Visit 2	Visit 3	DOF	F Value	*p* Value
**Glu&Gln**	**5.2 ± 0.6**	10.37 ± 1.05	10.16 ± 0.94	9.66 ± 1.16	**2, 35**	**7.34**	**0.002**
**Glu**	**5 ± 0.5**	7.5 ± 0.61	7.44 ± 0.69	7.19 ± 0.72	**2, 35**	**4.82**	**0.014**
**Gln**	**14.5 ± 1.9**	2.87 ± 0.6	2.72 ± 0.39	2.53 ± 0.52	**2, 34**	**4.15**	**0.024**
AcAc	32.3 ± 12.1	1.23 ± 0.15	1.16 ± 0.1	1.25 ± 0.14	2, 34	1.79	0.289
PCr	8.2 ± 1.3	0.18 ± 0.07	0.16 ± 0.04	0.16 ± 0.06	2, 35	1.29	0.583
BHB	38 ± 14.1	2.69 ± 0.33	2.65 ± 0.35	2.61 ± 0.34	2, 33	0.55	0.585
GSH	11.3 ± 1.7	0.35 ± 0.1	0.36 ± 0.1	0.36 ± 0.14	2, 33	0.55	0.739
NAA	2.2 ± 0.4	1.17 ± 0.16	1.17 ± 0.19	1.14 ± 0.18	2, 35	0.31	0.777
Ins	3.9 ± 0.4	6.96 ± 0.59	6.95 ± 0.38	6.87 ± 0.6	2, 35	0.25	0.847
GPC	3.3 ± 1.7	4.14 ± 0.42	4.09 ± 0.44	4.1 ± 0.43	2, 35	0.17	0.859
GPC&PCh	3.1 ± 0.3	1 ± 0.14	0.99 ± 0.14	0.97 ± 0.18	2, 35	0.15	0.893
Acn	58.5 ± 18.8	1 ± 0.14	0.99 ± 0.14	1 ± 0.16	2, 6	0.11	0.940
Cr	6.9 ± 1.4	0.05 ± 0.02	0.06 ± 0.02	0.06 ± 0.02	2, 35	0.06	0.960
NAA&NAAG	2.1 ± 0.3	3.07 ± 0.56	3.12 ± 0.5	3.09 ± 0.52	2, 35	0.04	0.986

Columns from left to right include the standard abbreviation for each metabolite, mean overall Cramer–Rao lower bounds and its standard deviation (SD), mean and SD for each water-scaled metabolite concentration for the three visits, degrees of freedom (numerator, denominator), F-value, and *p*-value. Statistical analysis involved repeated-measures linear mixed models with “Visit” as the repeated-measures variable and water-scaled concentration as the dependent variable. Additional factors and covariates are detailed in the Statistical Methods Section 2.9. Table is sorted by *p*-value, bold measures are significant for the omnibus test at *p* < 0.05.

## Data Availability

Datasets generated and/or analyzed during the current study are not publicly available but are available from the corresponding author on reasonable request.

## Supplementary Materials

The following supporting information can be downloaded at: https://www.mdpi.com/article/10.3390/cells1936224/s1. Figure S1. Cryo-EM; Supplemental Othor material S1: Study protocol; Supplemental Othor material S2: Scanner specific LCModel “control.txt” file.
