# Supplementary material for: Empagliflozin Induced Ketosis, Upregulated IGF-1/Insulin Receptors and the Canonical Insulin Signaling Pathway in Neurons, and Decreased the Excitatory Neurotransmitter Glutamate in the Brain of Non-Diabetics
Source: Cells. 2022 Oct 25;11(21):3372. doi: 10.3390/cells11213372 (PMC9657243; doi:10.3390/cells11213372)
Supplement: Supplementary file 1 [file cells-11-03372-s001.zip › Other material S1. Study protocol.pdf]

CLINICAL RESEARCH PROTOCOL

NATIONAL INSTITUTE OF HEALTH (NIH)
NATIONAL INSTITUTE ON AGING (NIA)

PROTOCOL NUMBER: 19AG0060 PROTOCOL VERSION: 1.3
TITLE: Sodium-glucose CoTransporter 2 (sGLT2) Inhibitor and Endogenous Ketone Production

SHORT TITLE: sGLT2 Inhibitor

IDENTIFYING WORDS: Alzheimer's Disease, ketones, hormones, metabolism

PRINCIPAL INVESTIGATOR: Josephine M. Egan, M.D., LCI, NIA, NIH

ESTIMATED DURATION OF STUDY: 3 Years

START DATE: 2018 END DATE: 2021

NUMBER AND TYPE OF PATIENTS: Accrual Ceiling: **100**

	Number	Sex	Age Range
Patients	N/A	N/A	N/A
Volunteers	20	M/F	55 and older

PROJECT USES IONIZING RADIATION: X None
PROJECT USES "DURABLE POWER OF ATTORNEY": N/A
OFF-SITE PROJECT: N/A
MULTI-SITE STUDY: N/A

Précis....5
1 Background Information and Scientific Rationale....6
1.1 Background and Rationale....6
1.2 Description of the Study....7
2 Study Objectives....7
2.1 Primary Objective....7
2.2 Secondary Objectives....8
3 Study Design....8
3.1 Description of the Study Design....8
3.2 Study Endpoints....8
4 Study Population....8
4.1 Rationale for Subject Selection....9
4.2 Recruitment Plan....9
4.3 Participant Inclusion Criteria....9
4.4 Participant Exclusion Criteria (Screening Visit)....9
4.5 Justification for Exclusion of Women, Minorities, and Children (Special Populations)....10
4.6 Justification for Inclusion of Other Vulnerable Subjects (NIH Staff)....11
5 Study Schedule....12
5.1 Phone Screening (Pre- Study)....12
5.2 Screening Visit....12
5.3 Visit 1....12
5.4 Visit 2....13
5.5 Visit 3....14
5.6 Follow-up Phone Call....13
5.7 Participant Withdrawal....15
5.8 Early Termination Visit....15
6 Study Procedures/Evaluations....15
6.1 Clinical Evaluations....16
6.2 Laboratory Evaluations....18
6.2.1 Laboratory Samples....18
6.2.2 Specimen Preparation, Handling and Shipping....19
7 Potential Risks and Benefits....20
7.1 Potential Risks....20
7.2 Potential Benefits....21
8 Research Use of Stored Human Samples, Specimens or Data....22
8.1 Storage....22
8.2 Tracking....22
8.3 Data....22
8.4 Disposition at the Completion of the Protocol....22
8.5 Reporting the Loss or Destruction of Samples/Specimens/Data to the IRB....22
9 Assessment of Safety....23
9.1 Event Characterization and Reporting to the IRB and the Clinical Director (CD)....23
9.2 Replacement of a Participant Who Discontinues Study Participation....23

10 Remuneration Plan for Subjects....23
11 Study Monitoring Structure....24
11.1 Site Monitoring Plan....24
11.2 Safety Monitoring Plan....24
12 Statistical Considerations....24
12.1 Participant Enrollment and Follow-Up....24
13 Ethics/Protection of Human Subjects....24
13.1 Responsibilites of Investigators…. 25
13.2 Protection of Vulnerable Subjects (Employees)…. 26
13.3 Informed Consent Process….27
14 Participant Confidentiality....26
15 Data Handling and Record Keeping....26
15.1 Data Management Responsibilities....26
15.2 Data Capture Methods....26
15.3 Source documents and Access to Source Data/Documents....27
Appendix A: Scientific References....28
Appendix B: Information Sheet for NIH Employees Participating in Intramural Research Protocols….31
Appendix C: Inpatient Food Diary….32
Appendix D: Discharge Instructions Visit 1….37
Appendix E: Intake Diary….38
Appendix F: Discharge Instructions Visit 2….40
Appendix G; Medication Log….41
Appendix H: Discharge Instructions Visit 3….43
Appendix I: Symptom Review….44
Appendix J: Study flyer….45

List of Abbreviations

AD	Alzheimer's Disease
AE	Adverse Event
ALT	Alanine transaminase
AST	Aspartate transaminase
ATP	Adenosine Triphosphate
ß-OHB	Beta-hydroxy butyrate
CBC	Complete Blood Count
CD	Clinical Director
CGM	Continuous Glucose Monitor
C-peptide	Connecting peptide
COPD	Chronic Obstructive Pulmonary Disease
CRC	Clinical Research Coordinator
DHHS	Department of Health and Human Services
EV	Extracellular vesicles
FDA	Food and Drug Administration
fMRI	Functional Magnetic Resonance Imaging
GABA	Gamma-Aminobutyric Acid
GCP	Good Clinical Practice
GNG	Gluconeogenesis
HbA1C	Hemoglobin A1C
HBsAG	Hepatitis B surface antigen
HIV	Human Immunodeficiency Virus
ICH	International Conference on Harmonization
I.V.	Intravenous
IRB	Institutional Review Board
IRP	Intramural Research Program
IRS	Internal Revenue Service
ISSO	Information System Security Officer
LCI	Lab of Clinical Investigation
LNS	Lab of Neurosciences
MAI	Medical Advisory Investigator
MCTs	Monocarboxylate transporters
mGluR	Metabotropic glutamate receptors
MRI	Magnetic Resonance Imaging
MRS	Magnetic Resonance Spectroscopy
(**mTOR**)	Mechanistic target of rapamycin
NAA	N-acetyl-L-aspartic acid
NIA	National Institute on Aging
NIDDK	National Institute of Diabetes, Digestive and Kidney Diseases
NIH	National Institutes of Health
NSAIDS	Non-Steroidal Anti-Inflammatory Agents
OHRP	Office of Human Research Protections
PI	Principal Investigator
R.N.	Registered Nurse
SAE	Serious Adverse Event
sGLT2	Sodium-Glucose CoTransporter2.
SOP	Standard Operation Procedure
VGLUT	Vesicular Glutamate Transporters

Précis

Objective and Specific Aims: The objective of this proof-of-concept study is to demonstrate in non-diabetic men and women age $\geq$ 55 years that a sGLT2 inhibitor will increase ketone bodies and metabolites used for gluconeogenesis. We also hypothesize that sGLT2 inhibitor (empagliflozin) will increase circulating glucagon and fatty acids, decrease circulating amino acids, increase expression of receptors and mediators of ketone metabolism in plasma exosomes and change Magnetic Resonance Spectroscopy (MRS) brain metabolism measures.

Experimental Design and Methods: 10 men and 10 women will be recruited for this pilot study. Each eligible participant will have a screen visit (Visit 0) and three additional 2-day study visits (Visit 1-3). On Visits 1, 2 and 3, frequent blood sampling for ß-hydroxybutyrate butyrate (ß-OHB), acetoacetate, fatty and amino acids, glucagon, insulin and glucose levels will be carried out; these visits will also include blood work for exosome markers and brain MRS. In addition, placement of a continuous glucose monitor (CGM) along with a 34-hour urine collection will be carried out. On Visits 1 and 2 the participants will wear the CGM until they return for their next visit. On Visit 3 the CGM will be removed at the end of the study visit. On Visit 1, no empagliflozin will be administered. Participants will return in 13 +/- 2 days for Visit 2. Visit 2 is the same as Visit 1 except empagliflozin 25 mg will be administered both mornings, at least 30 minutes before eating breakfast and participants will continue empagliflozin 25 mg once every morning, at least 30 minutes before eating breakfast, at home until they return in 13 +/- 2 days for Visit 3. At the end of Visit 3, empagliflozin will be stopped.

Medical Relevance and Expected Outcome: Elevating ketone bodies may bolster neuronal health and delay onset and progression of cognitive impairment. The expected outcome of this study is that we will see an increase in circulating levels of ketones, glucagon and fatty acids, an increased expression of receptors and mediators of ketone metabolism in plasma exosomes and a change in Magnetic Resonance Spectroscopy (MRS) brain metabolism measures, in subjects taking a sGLT2 inhibitor. We expect circulating amino acid levels will decrease, especially during the overnight hours. This study will aid in deciding whether this class of compound may be used in a larger study to improve cognitive function in patients with diagnosis consistent with declining cognitive function. We require that empagliflozin be taken for up to 2 weeks before returning for Visit 3, because we need to fully understand the homeostatic adaptations that may occur in the metabolite response to empagliflozin due to prolonged (up to 2 weeks) sGLT2 inhibition. It is our goal in the future to use the information gathered in this pilot study to design a long-term study in people who actually suffer from mild cognitive impairment/AD and therefore a Visit 2 (34-hour acute study) only, as outlined above, would not give us the full picture of the metabolic changes that might occur with prolonged use, especially in a non-diabetic population.

1 Background Information and Scientific Rationale

1.1 Background and Rationale

Cognitive decline and Alzheimer's disease (AD) are leading sources of morbidity and mortality in the aging population. In the U.S. there were an estimated 4.7 million individuals over the age of 65 with clinical AD and the number is projected to rise to 13.8 million by 2050 (10). Currently, there is no cure for dementia and no ways to delay its onset or slow its progression in humans Therefore, it is imperative that more research be focused on pathogenesis, pathophysiology and investigation of repurposing of drugs presently available so that treatments can be developed for managing dementia, and maybe at least preventing on-going neuronal functional decline. It is generally accepted that neurons use glucose as their primary source of energy. But neurons (as well as other cell types, such as heart muscle) can use ketone bodies (ß-OHB, acetoacetate) that are generated by the liver and transported to extrahepatic tissue as an energy source. There are elements of glucose dysregulation and decreased uptake in neurons of people with dementia, especially Alzheimer's disease (AD). The high metabolic efficiency of ketone bodies had implications for brain because ketone bodies can be an energy source when glucose availability is limited due to a pathological event, such as AD.

SGLT2 inhibitors are now approved treatment for type 2 diabetes since 2016. They lower blood glucose because they allow glycosuria to occur due to glucose not being resorbed from the collecting tubules. This leads to loss of glucose from the body, resulting in lower blood glucose and weight loss. Additionally, empagliflozin, in a large multicenter study, has been shown to be beneficial in patients with known cardiac disease.

SGLT2 inhibitors would be expected to increase endogenous ketone bodies (ß-OHB, acetoacetate) because when glucose is not resorbed back into circulation, glucagon levels in circulation become elevated and drive gluconeogenesis (GNG) in the liver. In order to provide substrate for GNG, lipolysis occurs, thereby supplying fatty acids for glucose and ATP formation. This should result in slightly increased ketone bodies in circulation, in particular during the overnight period. With this study, we wish to prove in the population who might benefit the most from increased endogenous ketone bodies whether this in fact occurs, and if it is sustainable – hence the need for at least the 14-day duration of treatment.

If SGLT2 inhibitors increase endogenous ketone bodies, then brain neurons and astrocytes may increase their metabolic reliance on ketone metabolism. This would be reflected in upregulation of monocarboxylate transporters (MCTs) required to import ketone bodies into the cell. MCT1 is not normally expressed in neurons or astrocytes, but it may be upregulated in astrocytes because of ketogenic diets. (https://www.proteinatlas.org/ENSG00000155380SLC16A1/tissue/cerebral+cortex#img) [1]. On the other hand, MCT2 and MCT4 are highly expressed in neurons and would be expected to be upregulated with SGLT2 inhibitors

(https://www.proteinatlas.org/ENSG00000118596-SLC16A7/tissue/cerebral+cortex#img; https://www.proteinatlas.org/ENSG00000141526-SLC16A3/tissue/cerebral+cortex#img).

We can probe the expression of these transporters in neurons and astrocytes using plasma exosomes enriched for neuronal and astrocytic origin as a source of biomarkers [2,3]. Other neuronal markers that mediate the effects of ketogenic diet in epilepsy and may change with sGLT2 inhibitors are the metabotropic glutamate receptor (mGluR) and VGLUT, which may also be probed with neuronal-origin exosomes. Finally, we can measure EV biomarkers reflecting the status the insulin – Akt – mTOR signaling pathway that determines cell energy metabolism [3]. These outcomes can be obtained through regular blood draws and shed light on the impact of the intervention on neuronal cellular metabolism.

Another methodology that can show changes in brain metabolism in response to SGLT2 inhibitors is brain Magnetic Resonance Spectroscopy (MRS). At the NIA 3T MRI Facility, we have the capacity to implement an advanced MRS method called J-PRESS. It can reliably detect previously elusive signals of important metabolites, such as glucose, lactate, ascorbate, as well as traditionally measured (but still important) metabolites, such as N-acetyl-aspartate (NAA) [4]. Applying this technique on a voxel within the bilateral precuneus, a region known for its susceptibility to AD pathogenic processes, we recently showed large and significant elevations in glucose and lactate and reductions in N-acetyl aspartate (NAA) in AD subjects compared to both older and younger controls [4]. In particular, NAA is a logical outcome for an intervention aiming to increase ketone availability and brain ketone utilization, since it was shown to increase in the hippocampus of mice consuming ketone esters [5]. In addition, we have shown that functional connectivity within brain networks is related to MRS levels of neurotransmitters [6]. To provide context and understand the impact of brain metabolic changes induced by SGLT2 inhibitors on brain function, resting fMRI will also be conducted. Any changes observed in these a priori hypothesized exosomal and MRS outcomes in this pilot study may be used to determine outcomes for future proof of concept studies of SGLT2 inhibitors in cognitive impairment and AD and inform the relevant power analyses.

1.2 Description of the Study

This pilot study is designed as a proof-of-concept study to examine the acute and chronic effects of a sGLT2 inhibitor (empagliflozin) on ketone body and metabolite production, as well as hormones directly involved in metabolism and nutrient utilization. This study will aid in deciding whether this class of compounds may be used for repurposing to improve cognitive function in patients with diagnosis consistent with declining cognitive function in a larger study.

2 Study Objectives

2.1 Primary Objective

To demonstrate that in non-diabetic humans, late middle-age to elderly, a sGLT2 inhibitor will increase ketone bodies, and metabolites used for GNG, especially during the night.

2.2 Secondary Objectives

- To determine the effects of such inhibition on a range of hormones and metabolites: insulin, glucagon, amino and fatty acid levels, especially overnight.
- To determine the effects of such inhibition on plasma exosomes biomarkers related to ketone metabolism (such as MCTs, receptors, intracellular mediators of insulin signaling and ketone metabolism).
- To determine the effects of such inhibition on brain MRS measures of neuronal integrity and metabolism (NAA, glucose, lactate).
- To investigate if this age group will tolerate this class of anti-diabetic drugs, for re-purposing.

3 Study Design

3.1 Description of the Study Design

This study involves a screening visit (Visit 0) and 3 study visits (Visits 1, 2 and 3) to the NIA Clinical Unit. In this pilot study, each enrolled participant will have a baseline visit (Visit 1) where no medication will be given and frequent blood draws x 34 hours for β-hydroxybutyrate butyrate (β-OHB), acetoacetate, fatty acids, glucagon, insulin and glucose levels will be done. Exosome studies, as well as brain MRS will also be done. The participant will have a continuous glucose monitoring system placed.

Visit 2 will take place in 13 +/-2 days from Visit 1. It will be the same as Visit 1, however, on Visit 2 participants will receive a daily (morning) oral dose of empagliflozin (Jardiance) 25 mg, followed by breakfast within 30 minutes. They will continue the daily dose of empagliflozin (Jardiance) 25 mg orally after discharge until they return for Visit 3 in 13 +/-2 days.

Visit 3 will be the same as Visit 2. At the end of Visit 3 the study drug will be discontinued, and the participant will have completed the study.

3.2 Study Endpoints

The primary study endpoint will be the level of circulating ketones on Visit 3 with a target of 1.5 times that of the participant’s baseline (Visit 1). Other outcomes will be secondary.

4 Study Population

Twenty participants, ten males and ten females, age 55 and older will complete this pilot study. The ceiling for this study (100) reflects the additional accrual that we may anticipate. If needed, the protocol will be amended to account for an increase in the number of participants needed to be recruited.

4.1 Rationale for Subject Selection

This is a pilot study. We will enroll non-diabetic men and women age 55 and older. This age group is at greatest risk of cognitive decline. Therefore, the safety of sGLT2 inhibitors needs to be assessed in this non-diabetic population.

4.2 Recruitment Plan

Subjects will be recruited using multiple strategies, including but not limited to advertising in newspapers, flyers, Internet, NIH and NIA websites, expos and health fairs, volunteers who have expressed interest in NIA studies, and word of mouth from other study participants. All participants are recruited based on the same inclusion and exclusion criteria.

4.3 Participant Inclusion Criteria

Inclusion Criteria:

- Age 55 years and older.
- Healthy (see exclusion criteria below).
- Able to understand the study risks and procedures, and consent to participate in the study.
- Able to read and speak English.

4.4 Participant Exclusion Criteria (Screening Visit)

Exclusion Criteria:

- History of diabetes (requiring any medical treatment other than diet and exercise) or fasting plasma glucose > 126 mg/dl or HbA1c> 6.5 %.
- History of hypoglycemia.
- BMI > 35 kg/m^2.
- Creatinine clearance less than 60 ml/min as measured by GFR.
- Glucosuria
- History of anemia within the past 6 months or Hgb <11.0 mg/dL for women and Hgb <12.5 mg/dL for men.
- Current steroid use or steroid use within 90 days of screening, excluding eye drops.
- Currently taking loop diuretics (Lasix, for example).
- Participant presently following a calorie restriction diet, low carb/high fat diet.
- HIV virus infection

- Hepatitis B infection, as evidenced by a positive HBsAG at screen visit.
- Hepatitis C infection that has not been treated. (The screen blood work must show HCV RNA quantitative is not detectable).
- Active infection/fever that may cause changes in glucose metabolism.
- Known allergy to sGLT2 inhibitors in the past.
- Thyroid dysfunction that is not controlled or treated. This will be determined by Free T3, T4, Free T4 or TSH not within MedStar Harbor Hospital laboratory normal ranges for this pilot study.
- Adrenal dysfunction as determined by a cortisol level not within the normal range for MedStar Harbor Hospital Laboratory for this pilot study.
- Kidney or liver disease, (GFR < 60 mL/min/1.73 m² and/or liver enzymes not within normal ranges for MedStar Harbor Hospital Laboratory for this pilot study.
- Severe gastrointestinal diseases such as Crohn's disease or ulcerative colitis requiring continuous treatment.
- History of severe pulmonary disease such as chronic obstructive pulmonary disease (COPD) or asthma requiring continuous medication use.
- Patients with known, or evidence of, peripheral vascular disease.
- History of chronic urinary tract infections.
- History of recurrent or recent dehydration in the past year.
- History of recurrent or recent vaginal yeast infection.
- Alcohol intake greater than 30 grams (drink more than 2 beers OR equivalent per day).
- History of severe psychiatric conditions associated with behavioral problems or requiring chronic medical treatment.
- Poor venous access.
- Inability to walk 2,000 steps
- Donation or loss of 400 mL or more of blood within 56 days prior to and subsequent to screening.
- Participation in another study in the past 30 days, in which a study drug was administered.
- Currently participating in another study unless the investigator feels it would not interfere with the study.
- History of a medical condition or any other reason that, in the opinion of the investigator, will make participation in this study unsafe.
- Blood work or urine tests that are not considered by the study physician to be in an acceptable range for the study.
- Metal implants and devices incompatible with 3T Magnetic Resonance Imaging (MRI), or another contraindication to MRI.

4.5 Justification for Exclusion of Women, Minorities, and Children (Special Populations)

Exclusion of Children:

This Pilot study is being specifically undertaken in a population at risk for cognitive decline, thus our recruitment of people 55 years or older.

Other Exclusionary Criteria:

- Participants with creatinine clearance less than 60 ml/min will be excluded due to investigating the effects on metabolites relevant to neuronal health. We do not want interference from alterations in metabolites due to renal insufficiency.
- Diabetics will be excluded because they are likely to have an increase in circulating ketones and a lowering of blood glucose while on sGLT2 inhibitors. We want to study if this will occur in non-diabetic subjects in the specified age group of interest.
- BMI > 35 kg/m^2 are excluded due to production and use of ketone bodies which are impaired in obese individuals (Vice E et al. Metabolism 2005).

Exclusion of Non-English-Speaking Subjects:

- The NIA Clinical Research Unit is located in Baltimore, Maryland. We do not have ready access to interpreters of different languages.

4.6 Justification for Inclusion of Other Vulnerable Subjects (NIH Staff)

We intend to include NIH staff (to include NIH contractors and special volunteers, guest researchers and trainees) in this study. The staff may through standard recruitment efforts screen for the study. No direct solicitation of employees/staff will take place. Participation will be voluntary and neither participation nor refusal to participate will have an effect either beneficial or adverse, on the participant's employment or position at NIH. *Informed Consent Process* and confidentiality maintained as per Section 13.3, *Subject Confidentiality,* Section 14, discussed below. All staff interested in enrolling in this study will be given a copy of the *NIH Information Sheet on Staff Research Participation* (Appendix B).

For NIH employees:

- Neither participation nor refusal to participate will have an effect, either beneficial or adverse, on the participant's employment or work situation.
- The NIH information sheet regarding NIH employee research participation will be distributed to all potential subjects who are NIH employees.
- The employee subject's privacy and confidentiality will be preserved in accordance with NIH policies, which define the scope and limitations of the protections.
- Subjects that are employees/staff will be consented in the usual manner. The inclusion of employees or staff is not anticipated to affect the research outcome; therefore, if the subject is a co-worker (including a supervisor, subordinate or coworker) the subject may be consented by another co-worker and/or

subordinate. The study PI will not be involved in the consenting of employees or staff.

- Remuneration for employees/staff will follow the leave policy for *NIH Employees Participating in NIH Medical Studies (Appendix B).*

5 Study Schedule

The study is expected to be completed by the end of 2021.

5.1 Phone Screening (Pre- Study)

Initially, potential participants are screened by the NIA recruiter using a standardized telephone screen. The PI, CRC or research nurses will review the telephone screen for completeness and contact the participant if there are any questions.

5.2 Screening Visit (Visit 0)

Participants who are eligible following the phone screen will come to the NIA Clinical Research Unit for about 1.5 hours. The screening visit will include the following tests and procedures:

a. Informed Consent.
b. Height/Weight and Vital Signs (blood pressure, pulse, temperature, respirations and pulse oximetry).
c. Fasting Blood Tests (fasting at least 8 hours): fasting glucose, Complete Blood Count (CBC), Liver and Kidney function, Thyroid function, Serum Chemistries, HbA1c, cortisol level, HIV, Hepatitis B surface antigen, and Hepatitis C antibodies.(If a participant has a positive Hepatitis C test, the MedStar Harbor Hospital Lab will automatically have further testing on the blood sample for HCV RNA quantitative). Positive HIV and Hepatitis testing results are reported to the Maryland Department of Health and the screened participant will receive counseling by the study medical staff.
d. Medical History and Physical Exam.
e. A snack or meal will be provided.

Based on the information collected, study eligibility is established, and the participant is invited to come for Visit 1. For eligible participants, a copy of their laboratory results will be provided prior to or at their Baseline Visit. If ineligible, a letter is sent within 1-3 weeks confirming ineligibility along with the results of their laboratory testing.

5.3 Visit 1 Baseline – No study medication given

At Visit 1, the enrolled participant will come to the NIA Clinical Research Unit for a two-day visit, lasting approximately 34 hours. The following procedures will be done:

a. Height/weight, orthostatic blood pressure measurements, pulse, temperature, respirations and pulse oximetry.

b. I.V. placement for fasting blood tests (fasting for at least 8 hours) including CMP, lipids, insulin and other hormones, and exosomes. Followed by blood sampling every 1.5-hours x 2, then q 1-hour x 1, then q 1.5-hours x 8, then q 2-hours x 4, then q 1.5-hours x 2, then q1-hour x 1, then q 1.5-hours x 4 (completes 34-hours). This frequent sampling will include β-hydroxybutrate (β-OHB), acetoacetate, fatty and amino acids, glucagon, insulin and glucose levels. On Day 2, exosomes will be repeated and oxysterols and lipidomics will be drawn.

c. Placement of a continuous glucose monitor (CGM) used to collect data for up to 14 days, worn by the participant until they return for Visit 2.

d. 34-hour urine collection for creatinine clearance, C-peptide, and other hormones.

e. Brain MRS and limited brain MRI for voxel placement, required anatomical studies and resting functional connectivity will be conducted.

f. All meals will be provided. The meals on visits 1, 2 and 3 will be pre-selected by the participant. Food intake will aim to be uniform at all visits and at consistent times. Participants will be asked to record the time and items they eat on a Food Diary (Appendix C). They will also be instructed to hydrate. This includes drinking 8 ounces of water every 4 hours except when asleep.

g. On the evening of Day 1, the participants will be given an activity monitor and will be instructed to walk 2,000 steps before bedtime.

h. Discharge instructions will be given (Appendix D). They will wear the CGM and be asked to complete an Intake Diary (Appendix E) to record consuming anything other than water to assist with the CGM data collection.

5.4 Visit 2 occurs 13 +/- 2 days from Visit 1

Initiation of sGLT2 inhibitor (empagliflozin)

At Visit 2, the enrolled participant will come to the NIA Clinical Research Unit for a two-day visit, lasting approximately 34 hours. This visit will be the same as Visit 1, except the participant will receive a daily dose of empagliflozin (Jardiance).

The following procedures will be done:

a. Height/weight, orthostatic blood pressure measurements, pulse, temperature, respirations and pulse oximetry.

b. I.V. placement for fasting blood tests (fasting for at least 8 hours) including CMP, CBC, lipids, insulin and other hormones, and exosomes. The study drug empagliflozin (Jardiance) will be administered at approximately 8:00 a.m. followed by blood sampling every 1.5 hours x 2, then q 1-hour x 1, then q 1.5-hour x 8, then q 2-hours x 4, then q 1.5-hours x 2, then q 1-hour x 1, then q 1.5-hours x4 (completes 34-hours). This frequent sampling will include β-hydroxybutrate (β-OHB), acetoacetate, fatty and amino acids, glucagon, insulin and glucose levels. On Day 2, exosomes will be repeated and oxysterols and lipidomics will be drawn.

c. Placement of a continuous glucose monitor (CGM) used to collect data for up to 14 days, worn by the participant until they return for Visit 3.

d. 34-hour urine collection for creatinine clearance, C-peptide, and other hormones.

e. Breakfast will be given within 30 minutes of dosing.
f. All meals will be provided. They will aim to be consistent items and times as what was consumed on Visit 1. Participants will be asked to record the time and items they eat on a Food Diary (Appendix C). They will also be instructed to hydrate. This includes drinking 8 ounces of water every 4 hours except when asleep.
g. On the evening of Day 1, the participants will be given an activity monitor and will be instructed to walk 2,000 steps before bedtime. They will be instructed when they are discharged to continue to walk 2,000 steps every evening while wearing the activity monitor. This will occur during the time they are taking the study medication at home.
h. Day 2: Participants will receive empagliflozin (Jardiance) 25 mg followed by breakfast within 30 minutes.
i. Brain MRS and limited brain MRI for voxel placement, required anatomical studies and resting functional connectivity will be conducted.
j. Following the final blood draw, participants will be sent home and instructed on self-administration of a daily morning dose of empagliflozin (Jardiance) 25 mg and will wear the CGM until returning for Visit 3.
k. Discharge instructions will be given (Appendix F). They will wear the CGM and be asked to complete an Intake Diary (Appendix E) to record consuming anything other than water to assist with the CGM data collection.
l. A staff member will phone participants daily to assure they are taking the study medication and to assess for any symptoms. We will also remind them about the importance to hydrate, as stated above (f).

5.5 Visit 3 occurs 13 +/- 2 days from Visit 2

At Visit 3, the enrolled participant will come to the NIA Clinical Research Unit for a two-day visit, lasting approximately 34 hours. The following procedures will be done:

a. Height/weight, orthostatic blood pressure measurements, pulse, temperature, respirations and pulse oximetry.
b. I.V. placement for fasting blood tests (fasting for at least 8 hours) including CMP, lipids, insulin and other hormones. The study drug empagliflozin (Jardiance) will be administered at approximately 8:00 a.m. followed by blood sampling the same as visits 1 and 2. Breakfast will be given within 30 minutes of dosing.
c. Placement of a continuous glucose monitor (CGM) used to collect data for up to 14 days, worn by the participant until the end of Visit 3.
d. 34-hour urine collection for creatinine clearance, C-peptide, and other hormones.
e. All meals will be provided. They will aim to be consistent items and times as what was consumed on Visits 1 and 2. Participants will be also be instructed to hydrate. This includes drinking 8 ounces of water every 4 hours except when asleep.
f. On the evening of Day 1, the participants will be given an activity monitor and will be instructed to walk 2,000 steps before bedtime.
g. Day 2: Participants will receive empagliflozin (Jardiance) 25 mg followed by breakfast within 30 minutes.
h. Brain MRS and limited brain MRI for voxel placement, required anatomical studies and resting functional connectivity will be conducted.

i. Following the final blood draw, participants will have completed the study. They will no longer take the study medication or wear the CGM.
j. Discharge instructions will be given (Appendix G).

5.6 Follow-up Phone Call

Participants will be contacted by a staff member within one week to assess whether they are having any symptoms.

5.7 Participant Withdrawal

Participants may decide to withdraw from the study at any point in time. Participants will be instructed to either write a letter or email their wishes to the Principal Investigator. The NIA will retain custody of the samples as outlined. The NIA is the exclusive owner of any data, discoveries or derivative materials from the sample materials and is responsible for the restriction of sample use at the participant's request. The samples collected in the study can be used by NIA investigators and their collaborators.

5.8 Early Termination Visit

The Principal Investigator may decide to take a participant off this study if it is believed to be in their best interest, if they fail to follow instructions, if new information becomes known about the safety of the study, or for other reasons the investigator believes are important. Participation in this study may be stopped at any time by the Principal Investigator without a participant's consent.

6 Study Procedures/Evaluations

Procedure	Screen	Visit 1	Visit 2	13 +/- 2 days post Visit 2	Visit 3
Informed Consent	X				
Review of Inclusion/Exclusion Criteria	X				
Medical History	X				
Physical Exam	X				
Height (cm) and Weight (kg)	X	X	X		X
Vital signs/orthostatic blood pressure measurements	X	X	X		X
CMP, ALT, AST, Alk. Phos., Total Bilirubin, CBC, Hepatitis B surface antigen, Hepatitis C, HIV and HbA1c, thyroid panel, cortisol level	X				
Urinalysis	X	X	X		X

Procedure	Screen	Visit 1	Visit 2	13 +/- 2 days post Visit 2	Visit 3
Blood samples for chemistry, lipids, oxysterols, lipodomics and exosomes (e.g., beta-amyloid)		X	X		X
CBC (Safety check)			X		X
Concomitant medication review	X	X	X		X
Insertion of venous catheter for frequent blood draws		X	X		X
Study medication (empagliflozin 25 mg daily)			X	X	X
Application of CGM		X	X		X
Diet Instructions / Record		X	X	X	X
Baseline blood and then frequent sampling (1.5-hours x 2, then q 1-hour x 1, then q 1.5-hours x 8, then q 2-hours x 4, then q 1.5-hours x 2, then q 1- hour x 1, then q 1.5-hours x 4. for β-hydroxybutrate (β-OHB), acetoacetate, fatty and amino acids, glucagon, insulin and glucose levels,		X	X		X
34-hour urine sampling for creatinine clearance, C-peptide and other hormones		X	X		X
MRS/MRI		X	X		X
Self-administration of study drug x 13 +/-2 days				X	
Meals/Snacks	X	X	X		X
Phone calls to monitor symptoms/compliance				X	
Adverse Event monitoring	X	X	X	X	X

6.1 Clinical Evaluations and MRI/MRS

Medical History and General Physical Examination

Participants receive a standardized physical exam and medical evaluation to include vital signs. Review of concomitant medications. This evaluation is required for study participation.

Body Measurements
Height and weight will be measured. These measurements are required for study participation.

Vital signs/Orthostatic blood pressures
This will be done to monitor for any problems of potential hypotension or dehydration.

Blood samples
Blood work for clinical chemistry, hematology, lipids, oxysterols, lipidomics, and exosomes will be done along with frequent blood sampling for hormones and ketones. An intravenous catheter will be placed for frequent sampling.

Safety Labs
Participants will have a fasting CMP/Lipid Panel on Day 1 of Visits 1, 2 and 3. On Visits 2 and 3 they will also have a CBC to monitor hemoglobin and hematocrit levels. Should a participant have a decrease of greater than 10% from the screening visit, the number of frequent blood draws would be decreased to every 2 hours. Therefore, the amount of blood taken on that visit would be approximately 130 ml (8.8 tablespoons).

Continuous Glucose Monitor (CGM)
The *Free Style Libre Pro* Continuous Glucose Monitor (CGM) device will be placed to measure interstitial glucose concentrations on Visits 1, 2 and 3. At the end of Visits 1 and 2 participants will wear the device until they return for Visits 2 and 3. The device will be removed at the end of Visit 3. The monitor consists of a small sensor that is inserted with a small needle and measures glucose levels just underneath the skin on the abdomen or the arm. The monitor will be inserted by the staff and the participant will be trained on wearing the device and who to call with questions or problems. A transmitter sends data wirelessly to the receiver. Once completed, data will be downloaded from the device to secure NIA servers.

Activity Monitor
On the evening of Day 1, participants will be given an activity monitor to wear on their wrist and will be instructed to walk approximately 2,000 steps. This will be done to try and keep activity levels similar in the 20 participants.

Study Medication
Empagliflozin (Jardiance) is an FDA approved drug used to treat type 2 diabetes. It is effective in lowering blood glucose levels in type 2 diabetic subjects, however, it is not known to lower blood glucose below normal levels (13). In other words, hypoglycemia is not a side-effect, it has not been reported to occur and is not therefore expected. An oral dose of 25 mg will be administered in the morning of days 1 and 2 of Visit 2. The participants will continue the study medication by self-administering for 13 +/- 2 days at home and recording on the medication log the time that they take their dose (Appendix G). The study medication will be discontinued at the end of Visit 3.

Diet Instructions/Records
Participants will be asked to record what they eat and drink during their inpatient visit on the Food Diary (Appendix C). They will be instructed on the importance of drinking 8 ounces of water every 4 hours, except when asleep. At discharge, they will be instructed to try and eat at the same times each day and record what time they eat or drink anything other than water on the Intake Diary (Appendix E).

Symptom Review Questionnaire
This will be used to assess the participant for any symptoms while taking the study medication. (Appendix I).

Compliance and symptom monitoring
Participants will be contacted daily between Visits 2 and 3 (during the days of self-administration of study drug) to ensure compliance and assess for any symptoms. We will also remind them about the importance of drinking water. It is imperative that participants do not miss 2 consecutive days of study medication or they will be excluded. The goal will be 100 percent compliance, however, greater than 90% will be acceptable.

Brain MRI: In total, structural MRI, fMRI and MRS will last about 1 hour.

- Structural MRI -- Standard structural brain images will be collected for coregistration, MRS voxel placement and to allow for clinical over-read.

- Resting state fMRI study in a single run. Subjects will be asked to keep their eyes open and fixate on a cross. This study will be used to assess intrinsic functional connectivity between different brain regions [6].

- MRS -- We will assess the concentrations of several brain metabolites, including glutamate, GABA, NAA, creatine, lactate, and glucose, using J-PRESS MRS in a voxel over bilateral precuneus, followed by fitting of metabolites using ProFit [6-8]. Each of these metabolites has a characteristic pattern of spectral lines defined by the energy of transitions that occur when nuclei are excited into a higher energy state through radio frequency pulses. The spectral lines from all metabolites present, taken together, make up the observed spectrum. Concentrations of individual metabolites can be determined through evaluation of the relative sizes of the spectral lines in the observed spectra. The reliability of the measurement and fitting procedure for each metabolite can be assessed using Cramer-Rao lower bounds [9]. We will use standard localization techniques based on magnetic field gradient sequences to obtain spectra from within the voxel of interest.

6.2 Laboratory Evaluations

6.2.1 Laboratory Samples

Blood Samples
Fasting blood samples are used to measure levels of chemicals, proteins, enzymes, inflammatory markers, markers of oxidative stress, lipids, minerals, hormones,

ketones and blood cells. Blood samples may include but are not limited to the following list.

Screen Visit: Fasting for at least 10 hours

- Comprehensive Chemistry Panel, ALT, AST, Alkaline Phosphatase, Total Bilirubin, thyroid panel
- Complete Blood Count (CBC) with Differential and Related Variables
- Hepatitis B surface antigen
- Hepatitis C antibody total
- HIV 1-2 antibody screen
- HbA1C
- Cortisol level
- Urinalysis

Visit 1

- Fasting Comprehensive Chemistry Panel, Lipids, oxysterols, lipidomics
- Exosomes (e.g., beta amyloid)
- β-hydroxybutrate (β-OHB), acetoacetate, fatty acids and amino acids
- Glucose, insulin and glucagon
- Urinalysis

Visits 2 and 3:

- Same as Visit 1, except CBC will be done for safety

<u>Urine Sample</u>
A 34-hour urine sample will be collected for creatinine clearance, C-Peptide and other hormones on Visits 1, 2 and 3.

6.2.2 Specimen Preparation, Handling and Shipping

Specimens are collected by research staff for clinical laboratory analysis and for research related to endocrinology and metabolism. No genetic testing will be performed.
All samples will be sent to the Core Lab via courier for pick-up by LCI staff.

Stored Samples: Samples of blood and tissue are stored in a deep freeze (-80 to -150˚C). Samples are labeled with the participant's study identification code. All specimens collected are assembled in a "biological bank" of samples that are used by NIA to address questions related to the aims of this study. Samples used for exosome studies will be pulled and thawed once immediately prior to exosome isolation and biomarker determination at LNS.

The stored samples are kept indefinitely, controlled and managed by the Principal Investigator and the NIA. Study information is kept private to the extent possible by the law. Use of the biological samples is limited to designated NIA investigators or their collaborators under the auspices of the NIA Scientific Director and the

authorization and supervision of the PI. Strict privacy about the samples is maintained.

7 Potential Risks and Benefits

7.1 Potential Risks

Risks of Blood Drawing and Intravenous (I.V.) insertion: The total amount of blood taken during a screening visit is up to 30 milliliters (ml) or 2 tablespoons. The total amount taken for Visits 1, 2 and 3 will be about 504 ml or 34 tablespoons. Participants may experience some discomfort and/or bruising at the site of needle entry. There is a remote chance of fainting or infection. The I.V. may need to be inserted if unable to obtain frequent blood samples.

Risk of Stored Samples: The greatest risk from the use of stored samples is the unplanned release of information from medical records. The chance that this information will be given to an unauthorized person without the participant's permission is very small. We will not enter any experimental information (research results) into any other medical record and we will not release information to a third party unless specifically authorized by the participant. Possible problems with the unplanned release of information include discrimination when applying for insurance and employment. Some problems may occur if a participant discloses information or he or she agrees to have his or her medical records released.

Risk of Hepatitis Virus B, hepatitis virus C, and human immunodeficiency virus (HIV) Testing: This study involves a number of blood tests that require the subject's blood be tested for blood-transmitted diseases such as hepatitis virus B, hepatitis virus C, and human immunodeficiency virus (HIV). If the participant is infected with HIV, hepatitis B (positive HBsAG) or C (HCV RNA quantitative is detectable), he/she will not be able to participate in this study. We will tell the subject what the results mean, how to find care, how to avoid infecting others, how we report these infections, and the importance of informing any partners of possible risk.

Risks of Study Medication empagliflozin (Jardiance): The most common adverse reactions associated with administration of empagliflozin are dehydration, dizziness, lightheadedness, weakness, yeast and urinary tract infections. To decrease these risks, participants will be encouraged to hydrate and to maintain good hygiene. Other side effects may include low blood sugar, nausea, and upper respiratory tract infections. There also may be other side effects that we cannot predict, including hypersensitivity reaction. Rarely, sudden episodes of kidney injury may occur. Also, the Food and Drug Administration (FDA) has issued a warning about rare occurrences of a serious infection of the genital area (necrotizing fasciitis of the perineum) associated with sGLT2 inhibitors. Both of these have only been known in people who have diabetes. We will monitor for any symptoms during our daily calls to the participant while they are on study medication. This will include questioning any symptoms of tenderness, redness,

or swelling of the genitals or the area from the genitals back to the rectum. We will also monitor for fever or general feeling of being unwell.

Risks of Continuous Glucose Monitor (CGM): Potential risks include a slight skin irritation from the adhesive.

Risks of Brain MRI and MRS: The Food and Drug Administration (FDA) has recently established safety criteria for human exposure to MRI studies. The MRI scanner used in this study satisfies these safety criteria. People are at risk for injury from the MRI magnet if they have pacemakers or other implanted electrical devices, brain stimulators, dental implants, aneurysm clips (metal clips on the wall of a large artery), metallic prostheses (including metal pins and rods, heart valves, and cochlear implants), permanent eyeliner, implanted delivery pump, or shrapnel fragments. Welders and metal workers are also at risk for injury because of possible small metal fragments in the eye of which they may be unaware. Participants will be asked to complete an MRI screening form for each MRI. If any participant is found not to be eligible for the MRI or refuse the MRI, they will be disqualified from the study. In addition, all magnetic objects (for example, watches, coins, jewelry, and credit cards) will be removed before entering the MRI scan room. Participants with history of surgeries, implanted devices, etc. will be asked to provide us with surgical reports mentioning the type of the clips, prostheses, etc. used. Every effort will be made to establish the MR compatibility or incompatibility of these implants, according to the safety standards of the NIA 3T MRI center, including obtaining reports by manufacturing companies for MR compatibility. The research staff will evaluate each person with a less severe history of claustrophobia on a case-by-case basis.
Regarding minor discomforts, radiofrequency pulses emitted by the MRI machine can induce mild muscle twitching in some participants. If the sensation becomes overly uncomfortable, the scan will be stopped. The loud sounds (> 120 dB) emitted by the MRI machine during scanning require earplugs but may cause mild acoustic discomfort.

7.2 Potential Benefits

This is not a treatment study. Participants can decide not to participate in the study. Participants do receive copies of laboratory tests within1-3 weeks after their study visit. These results do not replace tests completed by their primary care physicians. Copies of these tests will only be provided to their physicians after proper written release has been obtained.

Results that require immediate follow-up are communicated to the participant within 1 business day after the results become available to the staff, in person or by telephone. Information is provided to the participant’s physician if the participant consented to the release of information to the physician (completed the *Medical Release* form). We do not provide treatment for these conditions at NIH. The long-term benefit is that data and information gathered from this pilot will be used to design a treatment study in people with mild cognitive impairment/AD.

8 Research Use of Stored Human Samples, Specimens or Data

Intended Use: Specimens of blood, cells and other biological material are stored in a "biological bank" and used for this study only. Strict privacy about the samples is maintained.

8.1 Storage

Samples are kept at between -80 and -150˚C. Barcoded labels will contain the following information: the participant ID, the date and visit number of the sample collected and the specimen type. No other information is reported on the label. Samples are kept indefinitely, controlled and managed by the Principal Investigator (PI) and the NIA. The origin of the samples is kept private to the extent possible by the law. Use of the biological samples is limited to designated investigators under the auspices of the NIA Scientific Director and the authorization and supervision of the Principal Investigator. Official NIA collaborators may receive samples as part of a collaborative project with NIH.

8.2 Tracking

Tracking of samples is done through an electronic database that creates a correspondence between the information contained in the specimen label and its position in the specific freezer of the biological bank. All freezers are maintained by the NIA. Specimens are managed only by NIA authorized employees. Random quality control checks are conducted regularly.

8.3 Data

The NIA ISSO approved secure web-based study repository system will present electronic source documents and Case Report Forms (CRFs) for collecting and storing study data. The web-based study repository is password protected and maintained on the secure NIA/NIH Intranet with access limited to authorized NIA personnel only. Research use of stored study data can only be granted by the Study PI. with the appropriate documentation and approvals.

8.4 Disposition at the Completion of the Protocol

All samples in this pilot study will be stored in the NIA biological bank and will be maintained indefinitely.

8.5 Reporting the Loss or Destruction of Samples/Specimens/Data to the IRB

Regardless of the reason or circumstances, loss of any biological samples will be reported to the IRB.

- Any loss or unanticipated destruction of samples or data (for example, due to freezer malfunction) that compromises the scientific integrity of the data collected for the study; will be reported to the NIDDK IRB.
- Additionally, participants may decide at any point not to have their samples stored. In this case, the principal investigator will destroy all know remaining samples and report what was done to both the subject and the IRB. This decision will not affect the subject's participation in any other protocol at NIH.

9 Assessment of Safety

9.1 Event Characterization and Reporting to the IRB and the Clinical Director (CD)

Adverse events, non-compliance both serious or continuing, protocol deviations both major and minor, as well as unanticipated problems are defined and described by the NIH Office of Human Subjects Research Protection policy #801, and will be reported in accordance with this policy.

9.2 Replacement of a Participant Who Discontinues Study Participation

Participants lost to follow-up, drop out, or are unable to participant any longer will be replaced at the discretion of the Principal Investigator.

10 Remuneration Plan for Subjects

The amount of payment to research volunteers is guided by the National Institutes of Health policies. In general, participants are not paid for taking part in research studies at the National Institutes of Health. Meals will be provided to participants.

Participants will be compensated for research-related discomfort and inconveniences in accord with NIH guidelines. Compensation of $600 or more in one year will be reported to the IRS per federal regulation. The participants will be given 30 dollars at the Screening visit (Visit 0); The maximum amount allowable for the study is 1870 dollars, unless the study physician asks the participant to return to the NIA Clinical Unit for an unplanned visit. Then they will be paid an additional $20 for each unplanned visit.
The participant will receive the following for completing each of the study visit:

Screening (Visit 0)	30 dollars
Visit 1	540 dollars
Visit 2	600 dollars
Visit 3	700 dollars
Unplanned Visit	20 dollars

The breakdown of payments is as follows:

Frequent blood sampling	140 dollars
Continuous Glucose Monitor	150 dollars
Food Diary (in-patient)	20 dollars ($10/day)
Intake Diary (out-patient)	65 dollars
MRI	125 dollars
Activity Monitor/completing steps (in-patient)	25 dollars
34-hour urine collection	50 dollars
Study medication (self-dosing)	100 dollars

11 Study Monitoring Structure

11.1 Site Monitoring Plan

No data and safety monitoring board are required for this study. Data and safety will be monitored by the Principal Investigator.

11.2 Safety Monitoring Plan

All protocols at the NIA follow the NIA Data and Safety Monitoring Plan. This includes using the Level of Risk Assessment Monitoring Guidelines that has been established for the NIA following NIH rules and regulations to ensure good clinical practices in the conduct of clinical research. Participants will be informed about new information from this or other studies that may affect their health, welfare, or willingness to stay in this study.

12 Statistical Considerations

This is a pilot study therefore no power calculation can be done.

12.1 Participant Enrollment and Follow-Up

This is a pilot study of volunteers that consent to join the study. Potential participants who express the desire to be enrolled in the study are screened in the NIA Clinical Research Unit. Participants are enrolled once the informed consent is signed by the participant. Participants lost to follow-up, drop out, or are unable to participate any longer will be replaced at the discretion of the Principal Investigator.

13 Ethics/Protection of Human Subjects

Research conducted under this protocol will be conducted in compliance with the protocol, Good Clinical Practices (GCP) and all applicable regulatory requirements.

13.2 Protection of Vulnerable Subjects (Employees)

Protections for employees and staff participating in this study include 1) assuring that the participation or refusal to participate will have no effect, either beneficial or adverse, on the subject's employment or position at the NIH, 2) giving employees and staff who are interested in participating the "NIH Information Sheet on Employee Research Participation" prior to obtaining consent, and 3) assuring that there will be no direct solicitation of employees or staff.

This study collects sensitive information, i.e. collection of blood for HIV, Hepatitis B and C. The PI will train study staff regarding obtaining and handling potentially sensitive and private information about co-workers through staff discussions and written NIA procedures. Information pertaining to these sensitive blood results will be in the participant's NIH medical record. NIA procedures for medical records will apply for the protection of the employee's privacy and confidentiality.

13.3 Informed Consent Process

Informed consent is a process where information is presented to enable persons to voluntarily decide whether or not to participate as a research subject. It is an on-going conversation between the human research subject and the researchers about the essential information about the study, which begins before consent is given and continues until the end of the subject's involvement in the research. Discussions of essential information about the research will include the study's purpose, duration, experimental procedures, alternatives, risks, and benefits, and subjects will have the opportunity to ask questions and have them answered.

Written informed consent will be obtained from the participant prior to any screening visits, study procedures or treatments. The Principal Investigator or other designated qualified protocol investigators (listed on the protocol's face page) will explain the study in language understandable to the subject. Sufficient time and opportunity will be given for discussion of the research as well as to answer any questions they may have, taking care to minimize or eliminate the perception of coercion or undue influence. The participant and the investigator will sign the current IRB-approved informed consent document. A witness will also sign the consent document to attest only to the validity of the signature of the subject, not the validity or quality of the consent. A copy of the consent will be given to the subject for future reference. The signed documents will be placed in the subject's medical record .The consent process will additionally be documented in the medical record.
The rights and welfare of the participants will be protected by emphasizing to them that the quality of their medical care will not be adversely affected if they decline to participate in this study.

Participants that are employees/staff will be consented in the usual manner as noted above. Because this is a pilot protocol, the inclusion of employees or staff is not anticipated to affect the research outcome, therefore if the subject is a co-worker the

subject may be consented by another co-worker and/or subordinate. The study PI will not be involved in the consenting of employees or staff.

We do not plan or anticipate the enrollment of non-English speaking subjects in this pilot study. The NIA at this location does not have sufficient resources (interpreters) to assist with questions the participant may have during the informed consent process. Therefore, a short form consent will not be utilized.

Subjects may withdraw consent at any time throughout the course of the study.

14 Participant Confidentiality

All records will be kept confidential to the extent provided by federal, state and local law. The study monitors and other authorized representatives may inspect all documents and records required to be maintained by the Investigator, including but not limited to, medical records. Samples and data will be stored using codes assigned by the investigators. Records will be kept locked and all computer entry and networking programs will be done with coded numbers only. Clinical information will not be released without written permission of the subject, except as necessary for audit purposes by the IRB or OHSRP.

When results of an NIH research study are reported in medical journals or at scientific meetings, the people who take part are not named or identified. In most cases, the NIH will not release any information about a participant's research involvement without their written permission. However, if they sign a release of information form, for example, for an insurance company, the NIH will give the insurance company information from the participant's medical record. This information might affect (either favorably or unfavorably) the willingness of the insurance company to sell the participant insurance.

The Federal Privacy Act protects the confidentiality of a participants NIH medical record. However, participants should know that the Act allows for the release of some information from their medical record without their permission, for example, if it is required by the Food and Drug Administration (FDA), members of Congress, law enforcement officials, or authorized hospital accreditation organizations.

15 Data Handling and Record Keeping

15.1 Data Management Responsibilities

Electronic records will be kept using secure computer databases. These databases are password protected and maintained on a secure NIA/NIH Intranet with access limited to authorized NIA staff members. All NIA members who have access to these databases have the proper training on patient confidentiality as well as the required Human Subject Protection Training.

15.2 Data Capture Methods

The study will utilize both hard medical record copies/reports as well as electronic data. Our web-based application supports flexible data capture and reporting. The system is hosted at the NIA in Baltimore, MD. It is accessible via the Internet through the NIH firewall, on both Mac and Windows based computers. An Oracle relational database is utilized to capture and secure data entered through the web interface.

The study will be conducted with the protocol, DHHS, FDA, ICH and all applicable institutional, state and local requirements.

15.3 Source documents and Access to Source Data/Documents

Study data will be collected as noted above. The Principal Investigator is responsible for assuring that the data collected is complete, accurate, and recorded in a timely manner.

Appendix A: Scientific References

1. Forero-Quintero LS, Deitmer JW, Becker HM. Reduction of epileptiform activity in ketogenic mice: The role of monocarboxylate transporters. *Sci Rep.* 2017;7(1):4900.
2. Goetzl EJ, Schwartz JB, Abner EL, Jicha GA, Kapogiannis D. High complement levels in astrocyte-derived exosomes of Alzheimer disease. *Annals of neurology.* 2018;83(3):544-552.
3. Mustapic M, Eitan E, Werner JK, Jr., et al. Plasma Extracellular Vesicles Enriched for Neuronal Origin: A Potential Window into Brain Pathologic Processes. *Front Neurosci.* 2017;11:278.
4. Mullins R, Reiter D, Kapogiannis D. Magnetic resonance spectroscopy reveals abnormalities of glucose metabolism in the Alzheimer's brain. *Ann Clin Transl Neurol.* 2018;5(3):262-272.
5. Pawlosky RJ, Kemper MF, Kashiwaya Y, King MT, Mattson MP, Veech RL. Effects of a dietary ketone ester on hippocampal glycolytic and tricarboxylic acid cycle intermediates and amino acids in a 3xTgAD mouse model of Alzheimer's disease. *Journal of neurochemistry.* 2017;141(2):195-207.
6. Kapogiannis D, Reiter DA, Willette AA, Mattson MP. Posteromedial cortex glutamate and GABA predict intrinsic functional connectivity of the default mode network. *NeuroImage.* 2013;64:112-119.
7. Schulte RF, Lange T, Beck J, Meier D, Boesiger P. Improved two-dimensional J-resolved spectroscopy. *NMR Biomed.* 2006;19(2):264-270.
8. Schulte RF, Boesiger P. ProFit: two-dimensional prior-knowledge fitting of J-resolved spectra. *Nmr in Biomedicine.* 2006;19(2):255-263.
9. Bolliger CS, Boesch C, Kreis R. On the use of Cramer-Rao minimum variance bounds for the design of magnetic resonance spectroscopy experiments. *NeuroImage.* 2013;83:1031-1040.
10. Hebert LE, Weuve J, Scherr PA, Evans DA: Alzheimer disease in the United States (2010-2050) estimated using the 2010 census. Neurology 2013;80:1778-1783
11. Miller IJ: Variation in human fungiform taste bud densities among regions and subjects. The Anatomical Record 1986;216:474-482
12. Cheng LHH, Robinson PP: The distribution of fungiform papillae and taste buds on the human tongue. Archives of Oral Biology 1991;36:583-589
13. Zinman B, Wanner C, Lachin JM, et al. Empagliflozin, cardiovascular outcomes, and mortality in type 2 diabetes. N Engl J Med.2015; 373:2117–2128).
14. Al Jobori H, Daniele G, Adams J, Cersosimo E, Triplitt C, DeFronzo RA, Abdul-Ghani M. Determinants of the increase in ketone concentration during SGLT2 inhibition in NGT, IFG and T2DM patients. Diabetes Obes Metab. 2017 Jun;19(6):809-813.
15. Filippas-Ntekouan, S., Filippatos, TD., Elisaf, MS, SGLT2 inhibitors: are they safe? Oct 2017; 72-82.

Appendix B: **Information Sheet for NIH Employees Participating in Intramural Research Protocols**

Information Sheet for NIH Employees Participating in Intramural Research Protocols

As an NIH employee, contractor, IRTA or other associate, you may participate in Intramural research studies. You may be motivated by altruism, a commitment to research in your own or related fields, or want access to clinical trials of potential direct therapeutic benefit. When deciding, you should make an *informed* decision about participation. This information sheet offers some points to consider for employees who are considering research participation at NIH.

First, similar to any individual who is considering research participation, you should seek adequate information about the study purpose, what is required of you in terms of procedures, interventions and time, and what the potential risks and benefits are expected to be. For more information, see **http://www.cc.nih.gov/participate/studies.shtml**

Whether you are thinking about participation in a research study that is being conducted by your supervisor, or others who you work closely within your laboratory, branch, or unit, or research that is being conducted in an area for which you have no affiliation, you should consider some additional factors:

1. **Possible bias**. Are you confident that you can be unbiased about reporting answers, side effects, or other information that could influence the study outcome or risk to you?
2. **Confidentiality**. Are you comfortable sharing your medical history (including, for example, mental health history or sexually transmitted diseases) and your social history (e.g. substance use) with study investigators who may or may not be your coworkers, or with the possibility of them discovering something about your health during the study?
3. **Pressure**. Do you perceive any pressure or expectations from either the study team or your supervisor or colleagues regarding participation? If so, does that pressure influence your decision or make it difficult for you to choose whether or not to participate? Remember it is your choice whether or not to participate. If you have concerns about enrolling in a study, the Department of Bioethics and the Office of Human Subjects Research Protections (OHSRP) are both available to help.
4. **Time.** Can you take time off from work to complete the study requirements or participate solely during non-duty hours? See the NIH Policy Manual 2300-630-3 “Leave policy for NIH employees participating in NIH medical research studies”

According to NIH guidance, anticipated inclusion of employees in research studies must be approved by the IRB, and when the individual obtaining consent is a supervisor or co-worker of a prospective participant, an independent person (e.g. through the Clinical Center Department of Bioethics, National Institute of Mental Health Human Subjects Protection Unit, or other entity as approved by the Institutional Review Board (IRB)) should monitor the consent process, unless waived by the IRB.

If you have any questions or concerns, please contact the Department of Bioethics at (301) 496-2429 or the OHSRP at 301-402-3444.

Appendix C: Inpatient Food Diary

Sodium-glucose CoTransporter 2 (sGLT2) Inhibitor and Endogenous Ketone Production
Participant Inpatient Food Diary

Study I.D.: ____________ Date: ____________ Visit # ______

Time of Day	Approximate Amount (Wt. or Vol)	EXAMPLES<br>FOOD DESCRIPTION<br>(One item per line)
0600	8 FLUID OZ.	TROPICANA ORANGE JUICE WITH EXTRA PULP
	8 FLUID OZ.	COFFEE, REGULAR BREWED
	2 CUPS	KELLOGG'S CORN FLAKES CEREAL WITH…..
	1 CUP	SKIM MILK AND...
	1	MEDIUM BANANA
10:00 A.M.	2 OZ.	FAT FREE, UNSALTED PRETZELS, LABEL INCLUDED
	1 TBSP.	REGULAR MUSTARD
12:00 P.M.	1	TURKEY SANDWICH ON….
	2 SLICES	WHITE "WONDER" BREAD
	3 OZ.	SLICED DELI TURKEY, LOW SALT

Sodium-glucose CoTransporter 2 (sGLT2) Inhibitor and Endogenous Ketone Production
Participant Inpatient Food Diary

Study I.D.: ____________ Date: ____________ Visit # ______

Drinking water is very important. Please check box after drinking 8 ounces of water. Please drink at least (6) 8 ounces.

(1) □ (2)□ (3) □ (4) □ (5) □ (6)

DAY 1 – Page 1

Time of Day	Approximate Amount (Wt. or Vol)	FOOD DESCRIPTION (One item per line)

Sodium-glucose CoTransporter 2 (sGLT2) Inhibitor and Endogenous Ketone Production

Participant Inpatient Food Diary

Study I.D.: ____________ Date: ____________ Visit # ______

Time of Day	Approximate Amount (Wt. or Vol)	FOOD DESCRIPTION (One item per line)

DAY 1 – Page 2

Sodium-glucose CoTransporter 2 (sGLT2) Inhibitor and Endogenous Ketone Production
Participant Inpatient Food Diary

Study I.D.: ____________ Date: ____________ Visit # ______

Drinking water is very important. Please check box after drinking 8 ounces of water. Please drink at least (6) 8 ounces.
(1) □ (2)□ (3) □ (4) □ (5) □ (6)

**Time of Day**	**Approximate Amount (Wt. or Vol)**	**FOOD DESCRIPTION (One item per line)**

DAY 2 – Page 1

Sodium-glucose CoTransporter 2 (sGLT2) Inhibitor and Endogenous Ketone Production
Participant Inpatient Food Diary

Study I.D.: ____________ Date: ____________ Visit # ______

**Time of Day**	**Approximate Amount (Wt. or Vol)**	**FOOD DESCRIPTION (One item per line)**

DAY 2 – Page 2

Appendix D: Discharge Instructions Visit 1

- **Should you experience any problems with your I.V. site, notify the nursing staff. Mild tenderness and some bruising are not unusual.**
- **The Continuous Glucose Monitor will measure and store your sugar levels for up to 14 days. When you return for Visit 2, we will download the data which will show us how your sugar levels are trending throughout the day and night.**
  The sensor is comfortable enough to forget it is there. However, use care to avoid catching the sensor on clothing while getting dressed.
  Your sensor is water resistant and can be worn while bathing, showering or swimming as long as you do not take it deeper than 3 feet. Also, do not keep it under water for longer than 30 minutes at a time.
  Intense exercise may cause the sensor to loosen due to sweat or movement of the sensor. Please contact us if you have any skin irritation or discomfort or if the sensor should become loose or removed.
- **Try to eat the same times each day. Record what times you are eating or drinking on the log provided.**
- **Please drink plenty of fluids.**
- **Please do not donate any blood to the Red Cross during your study participation.**

Your next study appointment (Visit 2) is ____________________________________

Please arrive <u>fasting.</u> This visit will be the same as Visit 1 except you will be given the study medication.

Contacts:

**Denise Melvin, R.N.**	**410-350-3924**	**(Monday to Friday 6:30 am to 3 pm)**
**NIA Clinical Research Unit**	**410-350-3955**	**(Sunday 3 pm until Friday 5 pm)**
**Dr. Josephine Egan, M.D.**	**410-294-4759**	

If you are having a medical emergency, please call 911 and go to your local hospital for assistance.

Participant Signature: ____________________ Date: __________
Staff Signature: ____________________

Appendix E: Intake Diary

sGLT2 Inhibitor – INTAKE DIARY

PLEASE RECORD TIME YOU PUT ANYTHING INTO YOUR MOUTH,
<u>OTHER THAN WATER</u>

Day 1 - Date	Day 2 - Date	Day 3 - Date	Day 4 - Date	Day 5- Date	Day 6- Date	Day 7 - Date	Day 8 - Date	Day 9 - Date
Time hh:mm (am/pm)	Time hh:mm (am/pm)	Time hh:mm (am/pm)	Time hh:mm (am/pm)	Time hh:mm (am/pm)	Time hh:mm (am/pm)	Time hh:mm (am/pm)	Time hh:mm (am/pm)	Time hh:mm (am/pm)

IF YOU RUN OUT OF LINES, PLEASE USE THE BACK OF THIS FORM TO RECORD EATING/DRINKING

sGLT2 Inhibitor– INTAKE DIARY

PLEASE RECORD TIME YOU PUT ANYTHING INTO YOUR MOUTH, OTHER THAN WATER

Day 10 - Date	Day 11 - Date	Day 12 - Date	Day 13 - Date	Day 14- Date	Day 15- Date	Day 16 - Date
Time hh:mm (am/pm)	Time hh:mm (am/pm)	Time hh:mm (am/pm)	Time hh:mm (am/pm)	Time hh:mm (am/pm)	Time hh:mm (am/pm)	Time hh:mm (am/pm)

IF YOU RUN OUT OF LINES, PLEASE USE THE BACK OF THIS FORM TO RECORD EATING/DRINKING

Appendix F: Discharge Instructions Visit 2

- **Should you experience any problems with your I.V. site, notify the nursing staff. Mild tenderness and some bruising are not unusual.**
- **The *Continuous Glucose Monitor* will measure and store your sugar levels for up to 14 days. When you return for Visit 2, we will download the data which will show us how your sugar levels are trending throughout the day and night.**

  The sensor is comfortable enough to forget it is there. However, use care to avoid catching the sensor on clothing while getting dressed.
  Intense exercise may cause the sensor to loosen due to sweat or movement of the sensor. Please contact us if you have any skin irritation or discomfort or if the sensor should become loose or removed.
  Your sensor is water resistant and can be worn while bathing, showering or swimming as long as you:
    - **Do not take it deeper than 3 feet**
    - **Do not keep it under water for longer than 30 minutes at a time.**

  If necessary, notify security at the airport checkpoint that you are wearing the device. If you have an MRI or CT scan for medical purposes, you must remove your sensor prior to the procedure and notify us.
- **Try to eat the same times each day. Record what times you are eating or drinking on the log provided.**
- **Please drink plenty of fluids. Drink at least 8 ounces of water every 4 hours, except when asleep.**
- **Please maintain good hygiene to prevent urinary tract or yeast infections. Daily baths/showering recommended.**
- ***Study Medication:***
  It is important that you take the study medication (1 pill) the same time every morning followed by breakfast within 30 minutes.

A staff member will contact you to see if you are having any symptoms and to confirm you are taking the medication.

Your next study appointment (Visit 3) is ___________________________________

Please arrive fasting. This visit will be the same as Visit 2.

Contacts:

**NIA Clinical Research Unit**	**410-350-3955**	**(Sunday 3 pm until Friday 5 pm)**
**Denise Melvin, R.N.**	**410-350-3924**	**(Monday to Friday 6:30 am to 3 pm)**
**Josephine Egan, M.D.**	**410-294-4759**	

If you are having a medical emergency, please call 911 and go to your local hospital for assistance.

Participant Signature: _____________________ Date: __________

Staff Signature: _____________________

Appendix G: Medication Log

**Medication Log (Page 1 of 2)**<br><br>***** Remember to take study medication empagliflozin (Jardience) 1 pill every morning, followed by breakfast within 30 minutes**			
**DRINKING WATER IS VERY IMPORTANT**<br>**Please check box after drinking 8 ounces of water. Please drink at least (6) 8 ounces.**			
Day 1<br>___/___/___<br>Saturday	Time pill taken	Breakfast time	Comments/Symptoms<br><br>**WATER: 8 OUNCES**<br>**(1)** □ **(2)** □ **(3)** □ **(4)** □ **(5)** □ **(6)** □
Day 2<br>___/___/___<br>Sunday	Time pill taken	Breakfast time	Comments/Symptoms<br><br>**WATER: 8 OUNCES**<br>**(1)** □ **(2)** □ **(3)** □ **(4)** □ **(5)** □ **(6)** □
Day 3<br>___/___/___<br>Monday	Time pill taken	Breakfast time	Comments/Symptoms<br><br>**WATER: 8 OUNCES**<br>**(1)** □ **(2)** □ **(3)** □ **(4)** □ **(5)** □ **(6)** □
Day 4<br>___/___/___<br>Tuesday	Time pill taken	Breakfast time	Comments/Symptoms<br><br>**WATER: 8 OUNCES**<br>**(1)** □ **(2)** □ **(3)** □ **(4)** □ **(5)** □ **(6)** □
Day 5<br>___/___/___	Time pill taken	Breakfast time	Comments/Symptoms

Wednesday			**WATER: 8 OUNCES**<br>**(1) □ (2) □ (3) □ (4) □ (5) □ (6) □**
Day 6<br>___/___/___<br>Thursday	Time pill taken	Breakfast time	Comments/Symptoms<br><br>**WATER: 8 OUNCES**<br>**(1) □ (2) □ (3) □ (4) □ (5) □ (6) □**
Day 7<br>___/___/___<br>Friday	Time pill taken	Breakfast time	Comments/Symptoms<br><br>**WATER: 8 OUNCES**<br>**(1) □ (2) □ (3) □ (4) □ (5) □ (6) □**
Day 8<br>___/___/___<br>Saturday	Time pill taken	Breakfast time	Comments/Symptoms<br><br>**WATER: 8 OUNCES**<br>**(1) □ (2) □ (3) □ (4) □ (5) □ (6) □**

Medication Log (Page 2 of 2)

***** Remember to take study medication empagliflozin (Jardience) 1 pill every morning, followed by breakfast within 30 minutes**

DRINKING WATER IS VERY IMPORTANT
Please check box after drinking 8 ounces of water. Please drink at least (6) 8 ounces.

Day 9<br>___/___/___<br>Sunday	Time pill taken	Breakfast time	Comments/Symptoms<br><br>**WATER: 8 OUNCES**<br>**(1) □ (2) □ (3) □ (4) □ (5) □ (6) □**
Day 10<br>___/___/___<br>Monday	Time pill taken	Breakfast time	Comments/Symptoms<br><br>**WATER: 8 OUNCES**<br>**(1) □ (2) □ (3) □ (4) □ (5) □ (6) □**
Day 11<br>___/___/___<br>Tuesday	Time pill taken	Breakfast time	Comments/Symptoms<br><br>**WATER: 8 OUNCES**<br>**(1) □ (2) □ (3) □ (4) □ (5) □ (6) □**
Day 12<br>___/___/___<br>Wednesday	Time pill taken	Breakfast time	Comments/Symptoms<br><br>**WATER: 8 OUNCES**<br>**(1) □ (2) □ (3) □ (4) □ (5) □ (6) □**
Day 13<br>___/___/___<br>Thursday	Time pill taken	Breakfast time	Comments/Symptoms<br><br>**WATER: 8 OUNCES**

			**(1) □ (2) □ (3) □ (4) □ (5) □ (6) □**
Day 14<br>___/___/___<br>Friday	Time pill taken	Breakfast time	Comments/Symptoms<br><br>**WATER: 8 OUNCES**<br>**(1) □ (2) □ (3) □ (4) □ (5) □ (6) □**
Day 15<br>___/___/___<br>Saturday	Time pill taken	Breakfast time	Comments/Symptoms<br><br>**WATER: 8 OUNCES**<br>**(1) □ (2) □ (3) □ (4) □ (5) □ (6) □**
Day 16<br>___/___/___	Time pill taken	Breakfast time	Comments/Symptoms<br><br>**WATER: 8 OUNCES**<br>**(1) □ (2) □ (3) □ (4) □ (5) □ (6) □**

Appendix H: Discharge Instructions Visit 3

- ❖ **Should you experience any problems with your I.V. site, notify the nursing staff. Mild tenderness and some bruising are not unusual.**
- ❖ **If you have any questions or symptoms in the next few days, please give us a call.**
- ❖ **A staff member will contact you within one week to check if you are having any symptoms.**

Contacts:
NIA Clinical Research Unit
410-350-3955 (Sunday 3 pm until Friday 5 pm)

Denise Melvin, R.N.
410-350-3924 (Monday to Friday 6:30 am to 3 pm)

Dr. Josephine Egan, M.D.
410-294-4759

Participant Signature: ______________________   Date: ___________

Staff Signature: ______________________

Appendix I: Symptom Review

I.D.# - ___ ___ ___   Date: _____________   Time: ________   Visit # ______

Do you have any of the following symptoms?

Symptom	Yes	No	Comment
Dizziness			
Lightheadedness			
Weakness			
Yeast infection (i.e. itching, burning, redness, smelly discharge from genital area)			
Genital infection: tenderness, pain, swelling, flu-like symptoms			
Burning on urination			
Nausea			
Vomiting			
Diarrhea			
Constipation			

Confusion			
Feeling shaky			
Increased hunger			
Decreased hunger			
Headache			
Irritability			
Palpitations			
Anxiety			
Coughing			
Sneezing			
Nasal congestion			
Runny nose			
Fever			
Sore throat			
Shortness of breath			
Other			

Appendix J: Study Flyer

sGLT2 Inhibitor Pilot Study

This study is being done to give a diabetic medication to people that are not diabetic to see if it will increase ketones in their blood.

YOU MAY BE ELIGIBLE IF:

- You are healthy
- 55 or older
- No history of Diabetes

This study will take place at the NIA Clinical Research unit in Baltimore. This study requires 4 visits. 1 screening visit and 3 visits lasting 34 hours.

Participants will have blood testing, urine collection, and glucose monitoring.

FOR MORE INFORMATION PLEASE CALL:

410-350-3941

[illegible]
