# Supplementary figures and images for: Empagliflozin Induced Ketosis, Upregulated IGF-1/Insulin Receptors and the Canonical Insulin Signaling Pathway in Neurons, and Decreased the Excitatory Neurotransmitter Glutamate in the Brain of Non-Diabetics
Source: Cells. 2022 Oct 25;11(21):3372. doi: 10.3390/cells11213372 (PMC9657243; doi:10.3390/cells11213372)

**Figure S1. Cryo-EM**

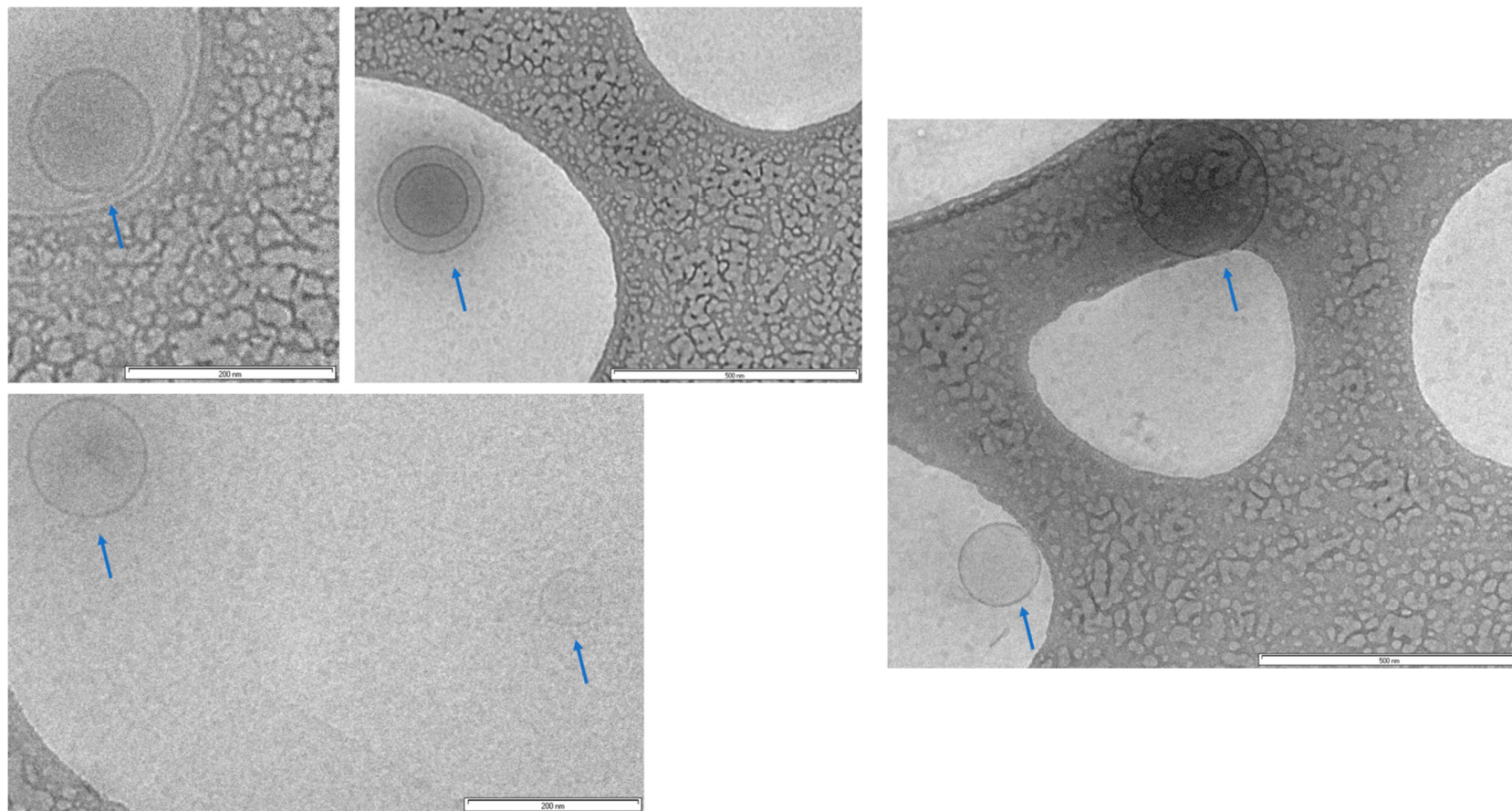

Supplement: Supplementary file 1 [file cells-11-03372-s001.zip › Figure S1. Cryo-EM.pdf]
